# Integrated network pharmacology and metabolomics to reveal the mechanism of QiShenYiQi Dripping Pills against cardiac structural and functional abnormalities

**DOI:** 10.3389/fphar.2022.1017433

**Published:** 2022-10-24

**Authors:** Jun Zhang, Zunyuan Yang, Xue Jia, Xinxin Li, Xiangyang Wang, Hua Rong, Yinan Liang, Wen Zeng, Wei Jia, Xiaohui Ma

**Affiliations:** ^1^ The State Key Laboratory of Core Technology in Innovative Chinese Medicine, Tasly Academy, Tasly Holding Group Co., Ltd, Tianjin, China; ^2^ PriMed Non-Human Primate Research Center of Sichuan PriMed Shines Bio-Tech Co., Ltd., Ya’an, Sichuan, China; ^3^ Institute of Pharmaceutical Sciences, China Pharmaceutical University, Nanjing, China; ^4^ Center for Translational Medicine and Shanghai Key Laboratory of Diabetes Mellitus, Shanghai Jiao Tong University Affiliated Sixth People’s Hospital, Shanghai, China; ^5^ School of Chinese Medicine, Hong Kong Baptist University, Hong Kong, China

**Keywords:** heart failure, QSYQ, SHR, pharmacology network, metabolomics

## Abstract

**Background:** Heart failure (HF), the final stage of cardiovascular diseases, is a clinical syndrome of cardiac structural or functional abnormalities. QiShenYiQi Dripping Pills, short for QSYQ, showed effectiveness and safety in the treatment of HF according to modern pharmacological research and clinical studies, but the mechanism remains unclear. This study aims to clarify the mechanism of QSYQ in treating heart failure through the analysis to critical biomarkers, targets and pathways.

**Materials and Methods:** In this study, the efficacies of QSYQ in non-human primates and rodents were evaluated, and the mechanism was demonstrated by integrating network pharmacology and metabolomics analysis. Furthermore, the targets from network pharmacology and the metabolites from targeted metabolomics were jointly analyzed to screen the critical pathways.

**Results:** In rhesus monkeys with spontaneous chronic heart failure, nasogastric administration of QSYQ for 12 weeks caused profound improvement of systolic and diastolic function as evidenced by echocardiography detection. Consistently, QSYQ administration especially with higher dose lowered the blood pressure and improved the ventricular remodeling, collagen deposition and fibrosis markedly in Spontaneous Hypertension Rats (SHR) model. Computational prediction showed that QSYQ exhibited anti-HF effects possibly through HIF-1 signaling pathway, FoxO signaling pathway, TNF signaling pathway, PI3K-Akt signaling pathway and other enriched paths. Metabolomics analysis obtained 23 significantly altered metabolites, revealing that QSYQ significantly regulated the abnormal levels of fatty acids, carnitines, organic acids pyridines, nucleosides, which were mostly involved in myocardial energy metabolism related pathways.

**Conclusion:** Based on serum and myocardium metabolomics and network pharmacology, the present study revealed that the actions of QSYQ in treating HF depend on multi-components, multi-targets and multi-pathways.

## 1 Introduction

Cardiac dysfunction is associated with a complex spectrum of pathophysiological changes, including metabolic disorders, inflammation, cellular senescence, cell death, and fibrosis (Tanai and Frantz, 2015). According to the definition on clinical terms, heart failure (HF) is a complex progressive disease in which the heart cannot sustain the supply of oxygenated blood to the body (Tham et al., 2015). The final stage of cardiovascular diseases is a clinical syndrome of cardiac structural or functional abnormalities. Until now, this challenging condition does not respond to simple one-dimensional treatment. Thus, additional studies remain to be required to ascertain the efficacy, safety and mechanisms of action of new and existing treatments and more therapies are needed to improve heart function and the quality of life of HF patients.

Traditional Chinese Medicine (TCM) compound is formed under the compatibility principles of Chinese herbal medicinal formulae and customarily composed of multiple medicinal herbs with different pharmacological properties (Su et al., 2016). QiShenYiQi Dripping Pills, short for QSYQ, composing of *Astragalus membranaceus* (AM), *Salvia miltiorrhiza* (SM), *Panax notoginseng* (PN), and *Dalbergia odorifera* (DO), is approved by the State Food and Drug Administration of China in 2003 for treatment of coronary heart disease.

Modern pharmacological research and clinical studies have demonstrated its effectiveness in the treatment of HF. Recent studies verified that QSYQ enhanced the degree of myocardial fibrosis by inhibiting the TGF-β1/Smads pathway and exerted an obvious myocardial protective effect on CHF rats (Lu et al., 2019). Chang et al. showed that QSYQ has the inhibitory effects on ventricular remodeling, cardiac ischemia-reperfusion injury (Chang et al., 2019). A systematic review and meta-analysis from clinical studies have demonstrated that QSYQ combined with conventional western medicine are better to improve the indicators of patients with CHF than conventional western medicine alone (Chen et al., 2021). Despite the aforementioned effects, the composition of TCM is too complex to systematically elaborate its mechanism of action.

**FIGURE 1:**
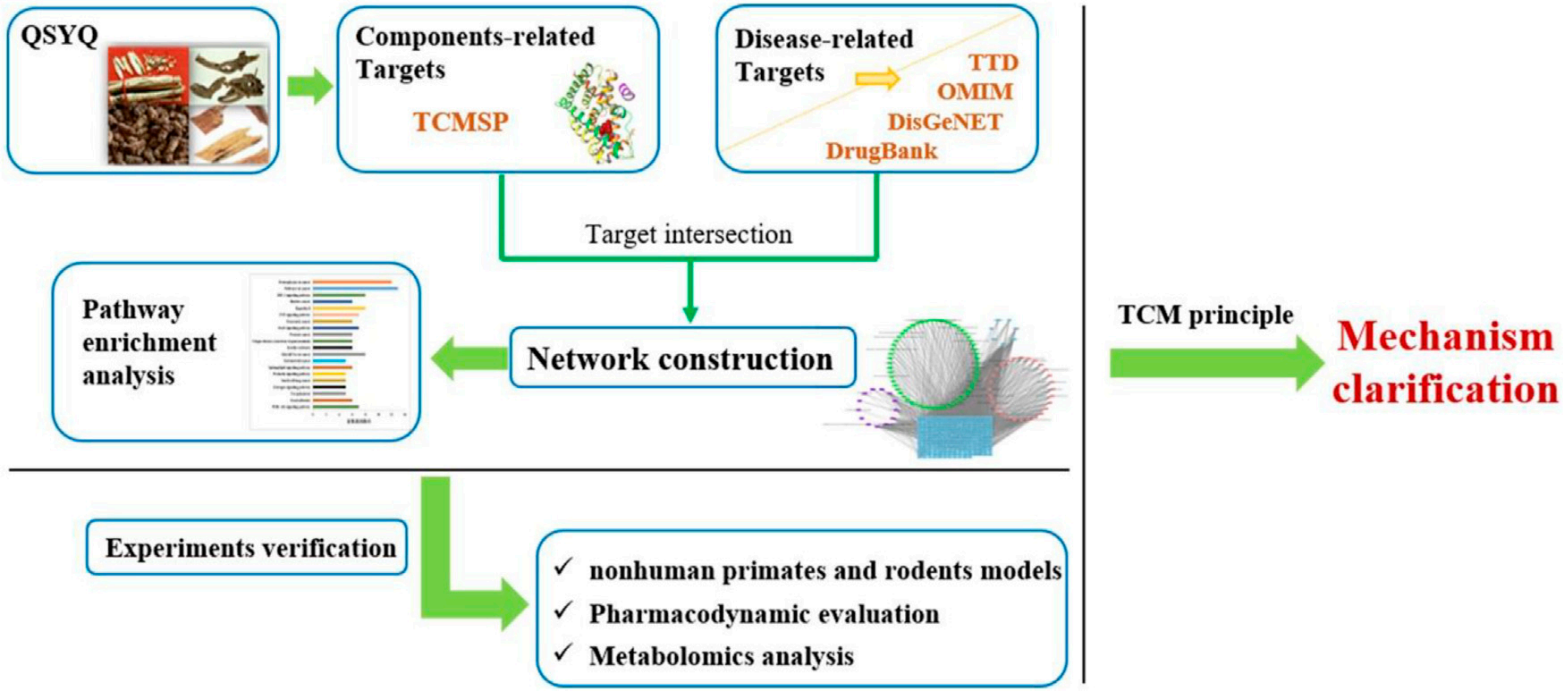
The flowchart of the study.

In this study, we reported the protective effect of QSYQ on rodent and non-human primate HF models. By integrating network pharmacology and metabolomics data acquired from rodents’ samples as well as reported results from publications, the molecular mechanism by which QSYQ exerts its pharmacodynamics effects was elucidated. The research flowchart is shown in Figure 1.

## 2 Methods and materials

### 2.1 Chemicals and reagents

QSYQ were provided from Tasly group, Tianjin with the batch No. For rhesus Monkeys and rats were 180615 and 190301, respectively and referred to commercial standards and quality control standards. Captopril (batch No. 1901001) as a control in this study was purchased from Tianjin Feiying Yuchuan Pharmacy Co., Ltd.

### 2.2 Network pharmacology analysis

As published (Ru et al., 2014), Traditional Chinese Medicine Systems Pharmacology database (TCMSP, https://old.tcmsp-e.com/tcmsp.php) could provide quantitative and systems information about TCM ingredients, ADME-related properties, targets and diseases. When retrieving the compound information of QSYQ, the screening criteria were set as the oral bioavailability (OB) greater than 30% and the drug-likeness (DL) greater than 0.18. It is worth mentioning that OB values were predicted by constructing a novel chemometric method, a SAR-based *in silico* models (Xu et al., 2012) while DL property was predicted from physicochemical properties of compounds (Shen et al., 2012). In addition, 10 major ingredients (Yunfei et al., 2008; Zhang et al., 2012; Shao et al., 2017) of QSYQ (as shown in Supplementary Table S1) were also included as active ingredients. Further targets prediction of the screened chemical ingredients and HF were achieved from databases like TCMSP and TTD (Hong et al., 2017), OMIM (Hamosh et al., 2005), DisGeNET (Janet et al., 2016), Drugbank (Wishart et al., 2018), PharmGkb (Whirl-Carrillo et al., 2012), respectively. UniProt database (https://www.uniprot.org/) was adopted to adjust the names of genes to official symbols while DAVID database (Huang da et al., 2009) to predict the related pathway. The results of network pharmacology and following experimental results were compared and integrated to illustrate the action mechanism of QSYQ.

### 2.3 Studies of rhesus monkey with spontaneous chronic heart failure

Male rhesus monkeys with spontaneous chronic heart failure (CHF) (Janet et al., 2016) were provided and housed in Sichuan Primed Shines Biotech Co. Ltd. Twelve monkeys from ages 13–23 years and with metabolic syndrome for more than 3 years were obtained based on meeting the following criteria conducted by GE Vivid S5 color Doppler ultrasound diagnostic instrument: diastolic dysfunction (Ea <8 or E/Ea > 8) or systolic dysfunction (LVEF: 20%–50%). The monkeys were individually housed and cared in a specific facility, which has been approved by the Association for the Assessment and Accreditation of Laboratory Animal Care International (AAALAC). They had free access to water and were fed *ad libitum* with Primed monkey chow (containing 18% protein, 62% carbohydrates, and 12% fat). All experimental protocols had been reviewed and approved by Institutional Animal Care and Use Committee (IACUC) of Sichuan Primed Shines Biotech Co., Ltd., with approval number AW 1813.

Animals were divided into QSYQ 50 mg/kg, QSYQ 100 mg/kg and vehicle group according to LVEF, Ea and E/Ea parameters detected in the adaptation period, each consisting of four monkeys. QSYQ and vehicle were orally administrated once a day (QD) for 12 weeks, followed by an additional 4-week washout period, cardiac function was evaluated every 4 weeks during this time.

### 2.4 Rat studies

Male SHR and WKY (*n* = 60, 14-week-old) were purchased from Beijing Vital River Laboratory Animal Technology Co., Ltd, Beijing, China. They were kept under a light/dark cycle of 12 h at controlled temperature and humidity. The rats were fed normally for 10 weeks and then randomly divided into five groups, each consisting of 13 rats. They were normal group (WKY), model group (SHR), captopril group (SHR + cap, 6.75 mg/kg), QSYQ low-dose group (SHR + LD, 155 mg/kg) and QSYQ high-dose group (SHR + HD, 465 mg/kg). As indicated, the rats were administrated daily by gavage with vehicle, captopril or QSYQ for 12 weeks. They were weighed once a week and the dosage for the next week was calculated accordingly.

The blood pressure of each rat was measured by KENT noninvasive sphygmomanometer while the cardiac function was evaluated by ultra-high resolution animal ultrasound imaging platform (VisualSonics^®^Vevo^®^3100) before administration, on the eighth week and after administration.

The rats were euthanized, then blood and tissues were collected immediately. Partial separated serum samples were used for metabonomic analysis. The tissues were weighed for HMI (heart mass index, heart weight/body weight) calculation, partial was adopted for histopathological analysis and partial for metabonomic analysis. All experimental protocols were approved by the local ethics committee of Tasly with approval number TSL-IACUC-2019-07. The relevant experiments were conducted in accordance with the Guidelines for the Animal Care and Use in Tasly.

### 2.5 Histologic analysis of cardiac tissues

For rats, hearts were fixed with 4% neutral paraformaldehyde solution for 48 h and embedded in paraffin wax. The heart sections (3–4 μm) were cut and mounted on glass slides for hematoxylin and eosin (H&E) staining and modified Masson staining. The histomorphology changes were observed under optical microscope.

### 2.6 Targeted metabolomics profiling

Targeted metabolomics profiling of serum and heart samples of rats were performed by Metabo-Profile (Shanghai, China, experimental details in Supplementary materials). Six rats were taken out and the left ventricle of myocardial tissue was quickly removed, separated and stored at −80°C. Based on ultra-high performance liquid chromatography tandem mass spectrometry (UPLC-MS/MS), the functional small molecule metabolites were quantitatively determined. Sample preparation and instrument parameter setting can be referred to published studies (Liu et al., 2020).

For statistical analysis, both principal component analysis (PCA) and orthogonal projection to latent structures square-discriminate analysis (OPLS-DA) were performed. The V-plot of the OPLS-DA model was used to visualize metabolites, while the candidate biomarkers were screened through multivariate and univariate statistical analysis by Variable importance in projection (VIP) > 1, *p* < 0.05 and |log_2_FC|>0 (Fold Change). Moreover, metabolic pathway analysis was performed by MetaboAnalyst 4.0 (https://www.metaboanalyst.ca/) to reveal disturbed metabolism. Pathways with the values of *p* < 0.05 were screened out as the candidate target pathways.

### 2.7 Statistical analysis

The measurement data for monkeys and rats were expressed as mean ± standard deviation (SD), unless otherwise stated. One-way analysis of variance (ANOVA) was used to analyze significant differences. A value of *p* < 0.05 was considered to be statistically significant.

## 3 Results

### 3.1 Network pharmacology analysis

**FIGURE 2:**
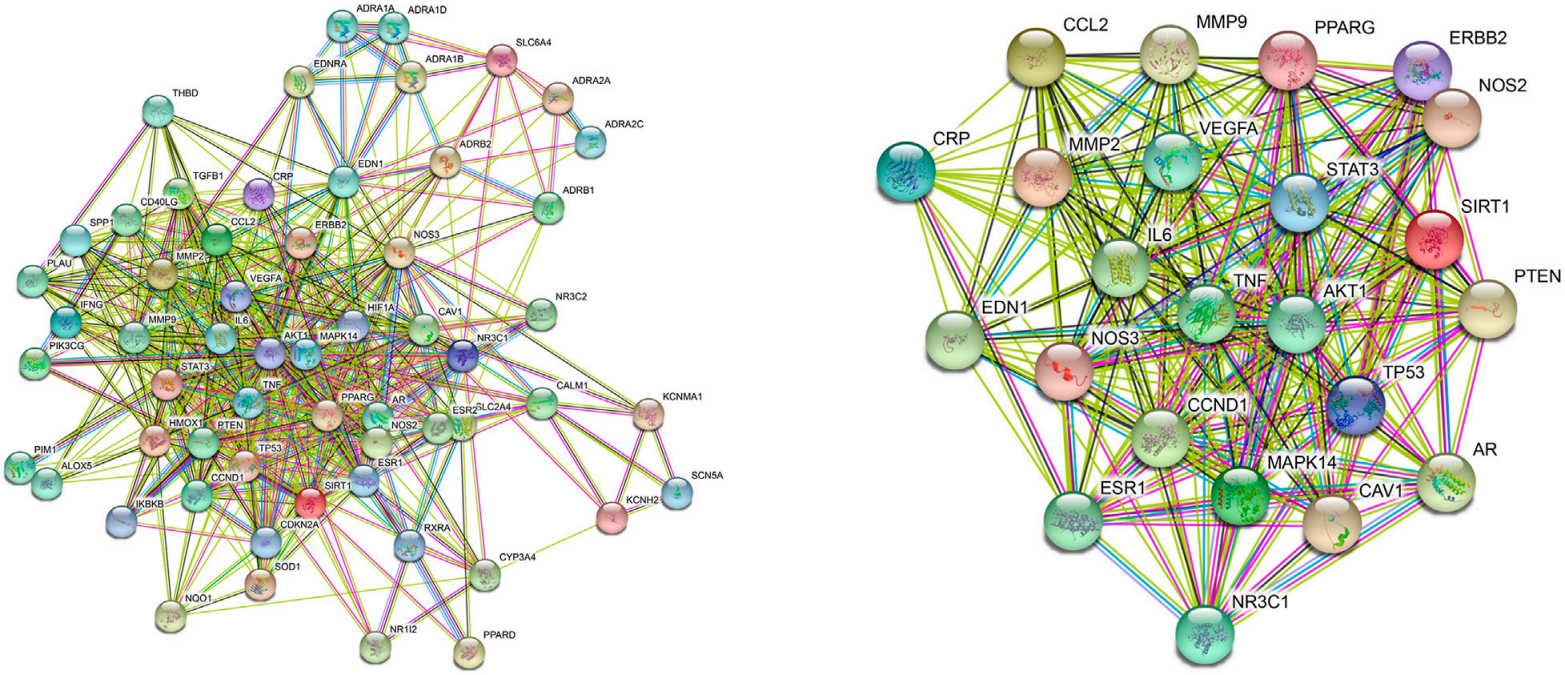
PPI network of the target intersection from QSYQ main chemical component and chronic heart failure. Target intersection (Left), key targets (Right).

**FIGURE 3:**
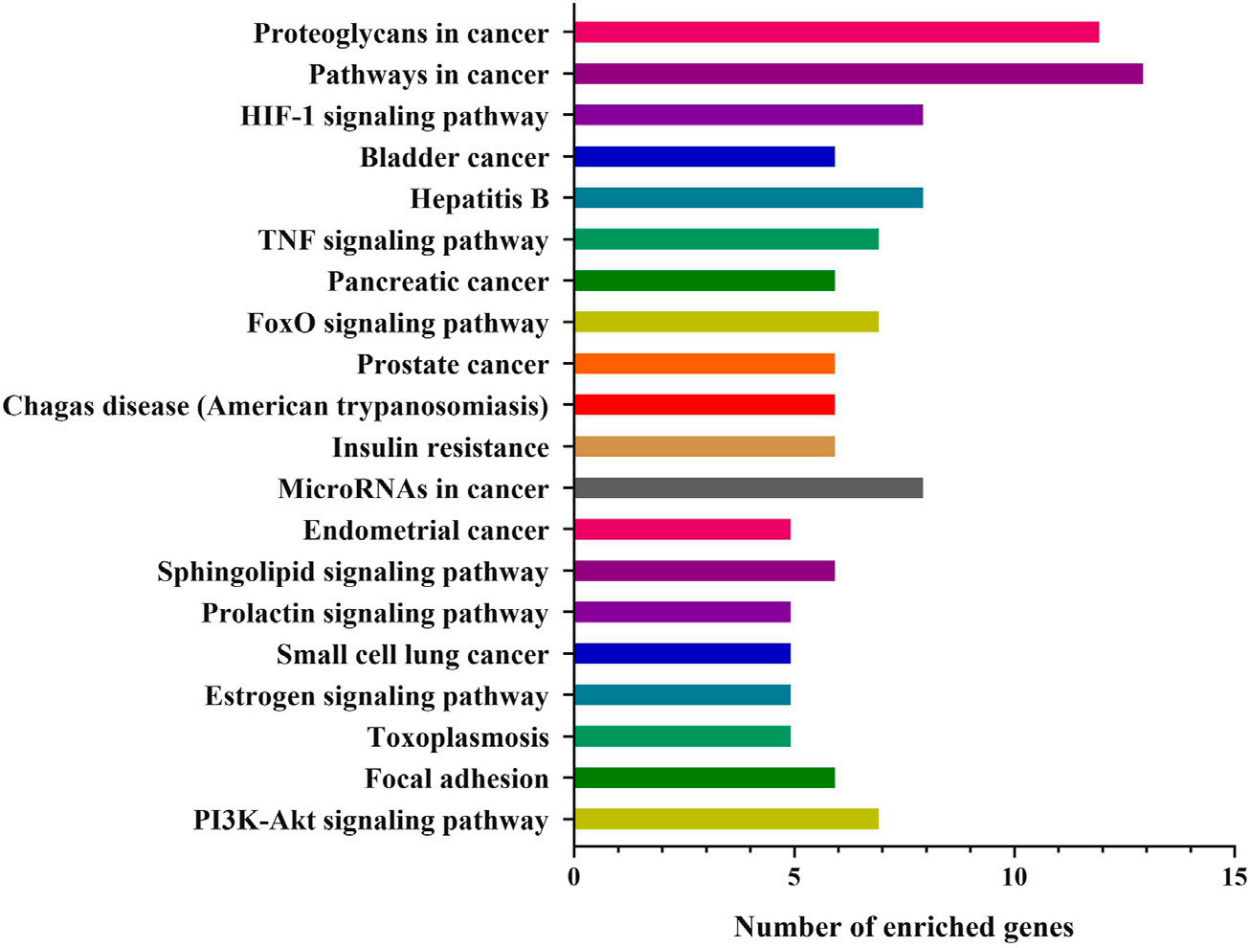
KEGG enrichment pathway analysis on potential targets from QSYQ against HF (top 20).

Network pharmacology’s comprehensiveness, systematicness and holistic concept are consistent with traditional Chinese medicine formula’s characteristic of multi-compound, multi-target and multi-pathway (Luo et al., 2020). Herein, Network Pharmacology was used to find the potential targets and signal pathways of QSYQ treating HF. DAVID and KEGG databases were combined for analysis of disease pathway enrichment analysis. The results showed that 131 potential active compounds were matched to 251 targets (Supplementary Tables S1, S2). Through comparison active ingredient targets to disease targets, 58 targets intersection of QSYQ against HF were received (Figure 2). According to the analytical results by Network Analyzer in Cytoscape software, 23 key targets (Figure 2) were used to enrich 42 disease pathways (Figure 3), including cancer-related pathways, HIF-1 signaling pathway, FoxO signaling pathway, TNF signaling pathway, PI3K-Akt signaling pathway (*p* < 0.05), which supported its critical role in the mechanism related to cardiovascular disease (Holscher et al., 2012; Xin et al., 2018).

### 3.2 QSYQ ameliorated the cardiac function in rhesus monkey

**FIGURE 4:**
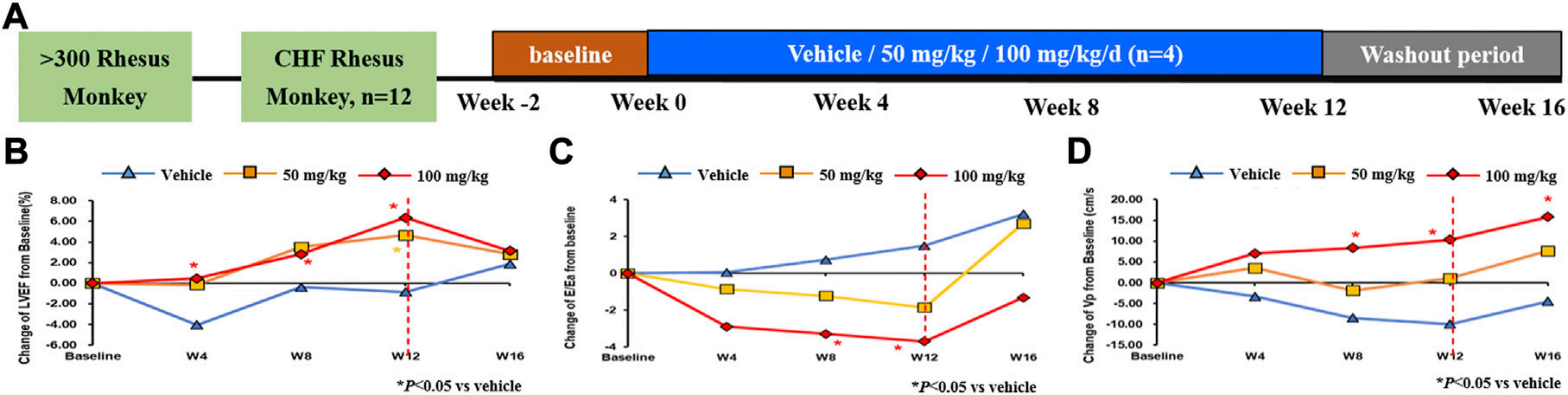
Administration of QSYQ ameliorated the cardiac function in rhesus monkey. Study design: 12 male rhesus macaque monkeys, which were initially screened from more than 300 monkeys, were treated with 50 mg/kg, 100 mg/kg or vehicle by nasogastric administration for 12 weeks, followed by an additional 4-week washout period **(A)**. The cardiac function was evaluated by LVEF index **(B)** for systolic function and E/Ea **(C)** and Vp **(D)** indicators for diastolic function. *, *p* < 0.05 vs. vehicle.

To investigate the role of QSYQ on the cardiac function, a study in rhesus monkeys with spontaneous chronic heart failure was conducted (Figure 4A). By Doppler echocardiography, an improved LVEF was observed in QSYQ treated monkeys compare with baseline level (Figure 4B). The elevated values were 4.67 ± 0.89% and 6.35 ± 4.79%, respectively for QSYQ 50 mg/kg and 100 mg/kg groups. Notably, compared to vehicle, the monkeys treated with 100 mg/kg QSYQ for 12 weeks showed a significantly increase in LVEF (*p* < 0.05). The LVEF improvement demonstrated time-dependent and dose-dependent during the administration period.

The velocity of annular motion reflects shortening and lengthening of the myocardial fibers along a longitudinal plane. As reported, Ea, which records the early diastolic velocity at the lateral corner of the annulus, is an index of LV relaxation. The ratio of early transmitral flow velocity (E) and Ea (E/Ea) can be adopted to evaluate the diastolic function if under normal state of systolic function (Nagueh et al., 1997). Similar decreasing trend of E/Ea were observed in monkeys that received QSYQ 50 mg/kg and 100 mg/kg with different time when compared to vehicle, while higher dose received much more evident action (*p* < 0.05) (Figure 4C). Left ventricular blood flow velocity in early diastole (Vp) is another index to judge the diastolic function. The results tended to increase and animals administered with 100 mg/kg improved further dramatically. (Figure 4D). Consistently, E/Ea, and Vp amelioration reflecting the diastolic function was time-dependent and dose-dependent.

### 3.3 QSYQ lowered the blood pressure in rats

**FIGURE 5:**
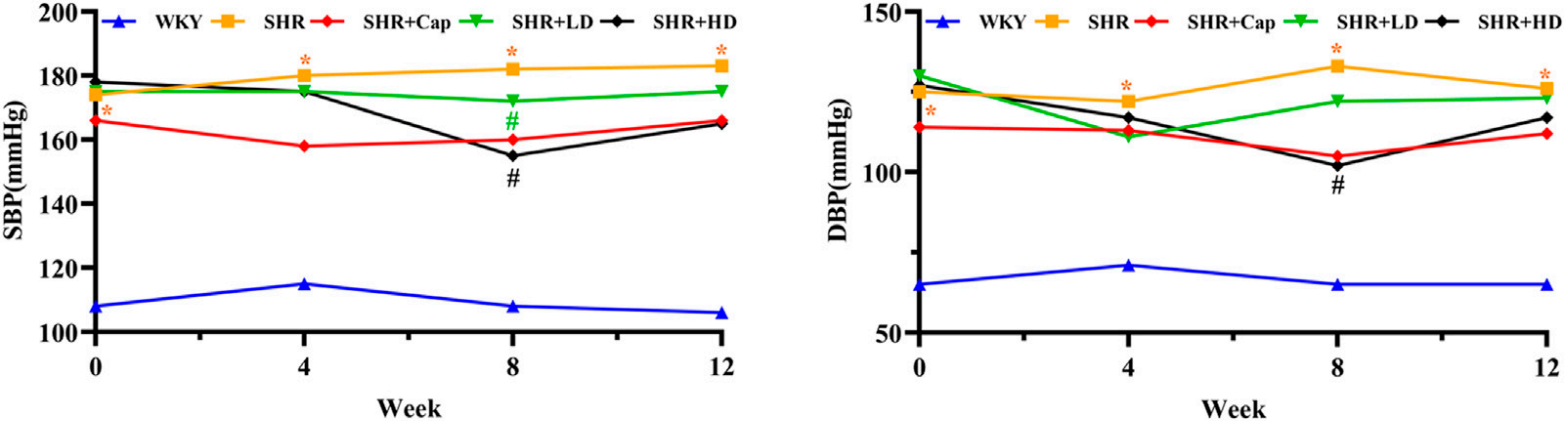
Blood pressure trend before and after treatment in each group. Values were presented as mean values, *n* = 13 per group. *, *p* < 0.05 vs. WKY. #, *p* < 0.05 vs. SHR. SBP, systolic blood pressure. DBP, diastolic blood pressure.

Hypertension is the leading risk factor for numerous cardiovascular diseases, and chronic pressure overload leads to the development of left ventricular hypertrophy (LVH). Progressive hypertrophy and fibrotic changes in the heart lead to progressive diastolic dysfunction ultimately leading to diastolic heart failure (Zhao et al., 2021). To further evaluate the effect of QSYQ on the blood pressure of SHR rats, systolic blood pressure (SBP) and diastolic blood pressure (DBP) were measured over the administration period. As shown in Figure 5, from the sixth week of QSYQ administration, SBP and DBP tended to decrease, which was significantly obvious in QSYQ high-dose group (SHR + HD, 465 mg/kg) compared to the model group.

### 3.4 QSYQ ameliorated the ventricular remodeling in rats

**FIGURE 6:**
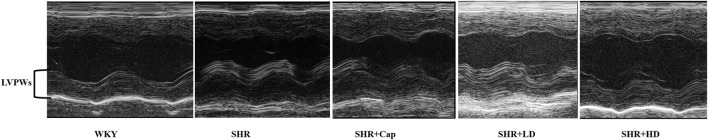
Representative echocardiograms of rat hearts in various conditions for determining the ventricle wall thickness (*n* = 13). LVPWs, left ventricular posterior wall (systole).

It has been verified that QSYQ could ameliorated the cardiac function in rhesus monkeys. To further prove the effects in rats, echocardiographic measurements were conducted correspondingly. Compared to WKY group (Figure 6, Table 1), the pressure over-loaded heart exhibited marked hypertrophy by parameters like LV Mass AW, LVPWs and heart mass index (HMI), which indicated the changes of cardiac remodeling. The alterations were significantly ameliorated through administration of QSYQ, especially after treating with high-dose QSYQ.

**TABLE 1:** The cardiac structure related parameters of rats after 12-week administration of QSYQ.

Groups	LV Mass AW (mg)	LVPWs (mm)	HMI (%)
WKY	1084 ± 155	3.39 ± 0.57	0.32 ± 0.042
SHR	1300 ± 228^*^	4.04 ± 0.44^*^	0.37 ± 0.016^*^
SHR + Cap	959 ± 144^**#**^	3.51 ± 0.34^**#**^	0.36 ± 0.013^#^
SHR + LD	1238 ± 223	3.70 ± 0.36^**#**^	0.35 ± 0.042
SHR + HD	1149 ± 110^**#**^	3.62 ± 0.35^**#**^	0.35 ± 0.019^#^

Values were presented as mean±*SD*, *n* = 13 per group. *, *p* < 0.05 vs. WKY. #, *p* < 0.05 vs. SHR. LV, Mass AW, the weight of left ventricle; LVPWs, left ventricular posterior wall (systole). HMI, heart mass index.

### 3.5 Administration of QSYQ protected the rats against collagen deposition and fibrosis

**FIGURE 7:**
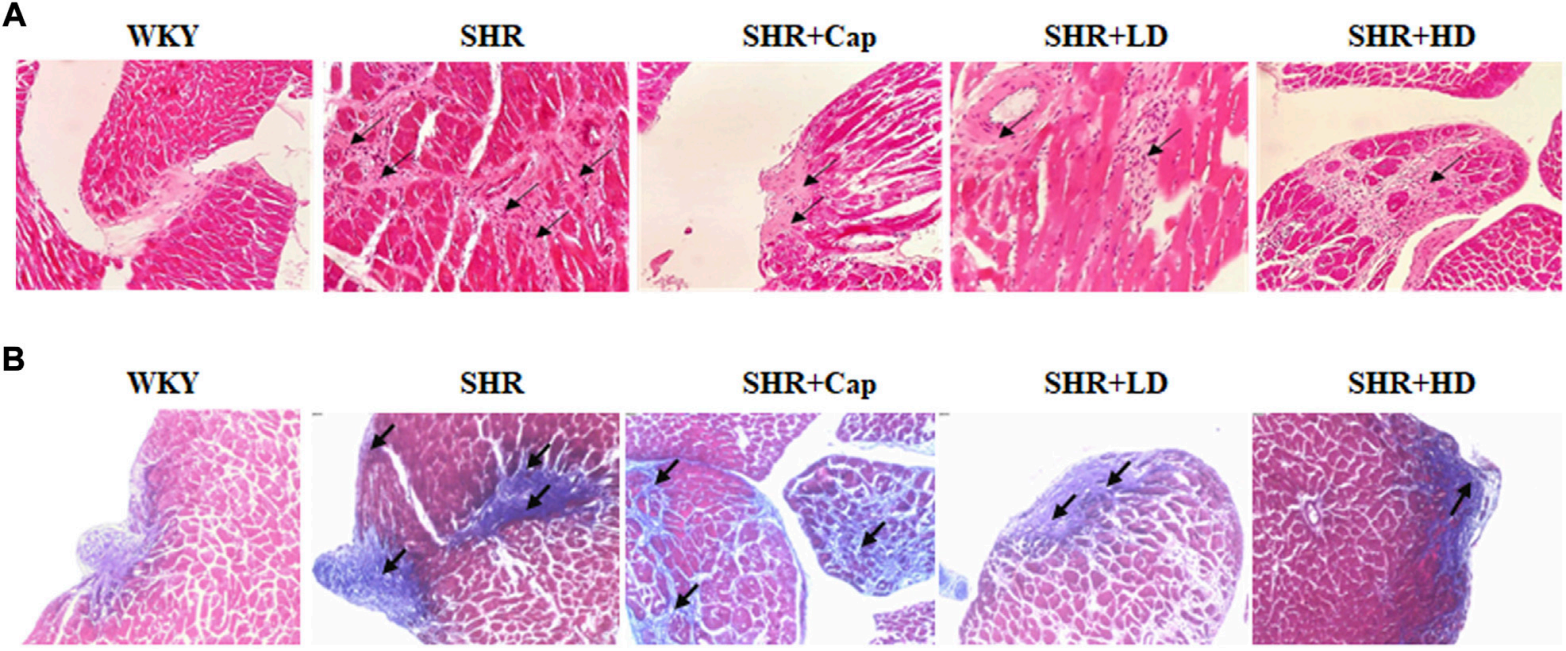
Administration of QSYQ protected the rat against collagen deposition and fibrosis. Representative histological images of H and E **(A)** and Masson **(B)** staining were shown.

Consistent with the above results, compared to SHR, diffused collagen deposition was improved after 12 weeks of QSYQ treatment by H&E staining (Figure 7A), although the dosage effects were not obvious. Moreover, masson staining revealed that QSYQ administration could reverse pericellular fibrosis, and the higher dose group seemed to improve significantly (Figure 7B). The cells in QSYQ-treated groups were arranged neatly and the morphology was normal, while disorganized cells and intercellular spaces were observed in the model group. These results indicated that QSYQ (T101) treatment could alter cardiac structure and prevent the progression of HF in rats.

### 3.6 QSYQ regulated the abnormality of serum and myocardial tissue metabolic profiles

When the heart is damaged, the myocardial structure changes as well as the metabolism of glucose, fatty acid, lactic acid and other substances in myocardial cells are disturbed, which will lead to the abnormality of cardiac function. Herein, based on the results of pharmacodynamics evaluation above, metabolomics was conducted to explore the discrimination of metabolite profiles among six samples from the WKY control, the SHR model and QSYQ high dose treatment groups, respectively.

**FIGURE 8:**
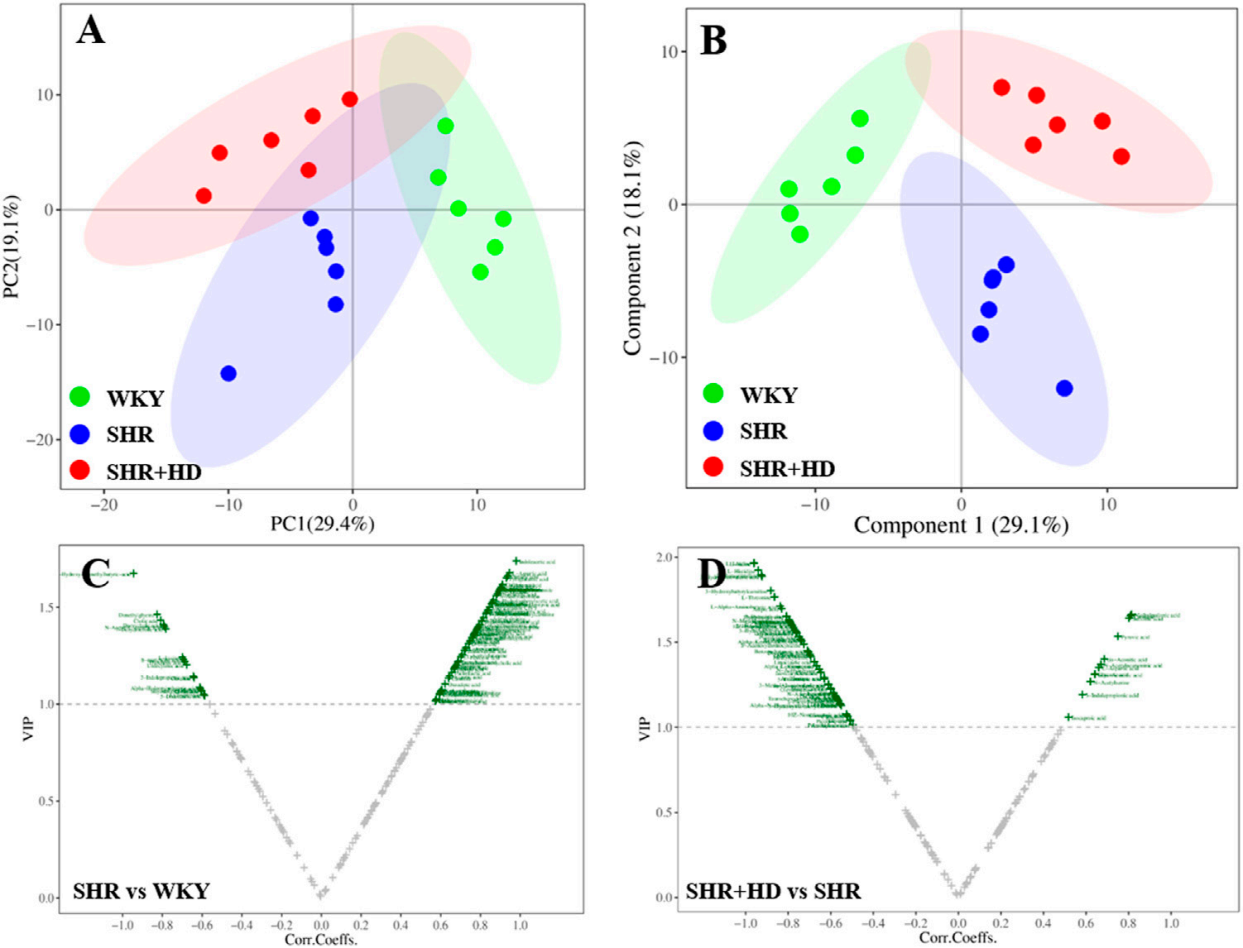
Multivariate statistical analysis of serum samples among WKY, SHR, and QSYQ high dose treatment groups. **(A)**, PCA score plot. **(B)**, PLS-DA score plot. **(C,D)**, VIP volcanic map of OPLS-DA model. The PCA and PLS-DA were performed on all groups while the OPLS-DA was performed between two groups. PCA, principal component analysis, PLS-DA, partial least squares discriminant analysis, OPLS-DA, orthogonal projection to the latent structure with discriminant analysis.

**FIGURE 9:**
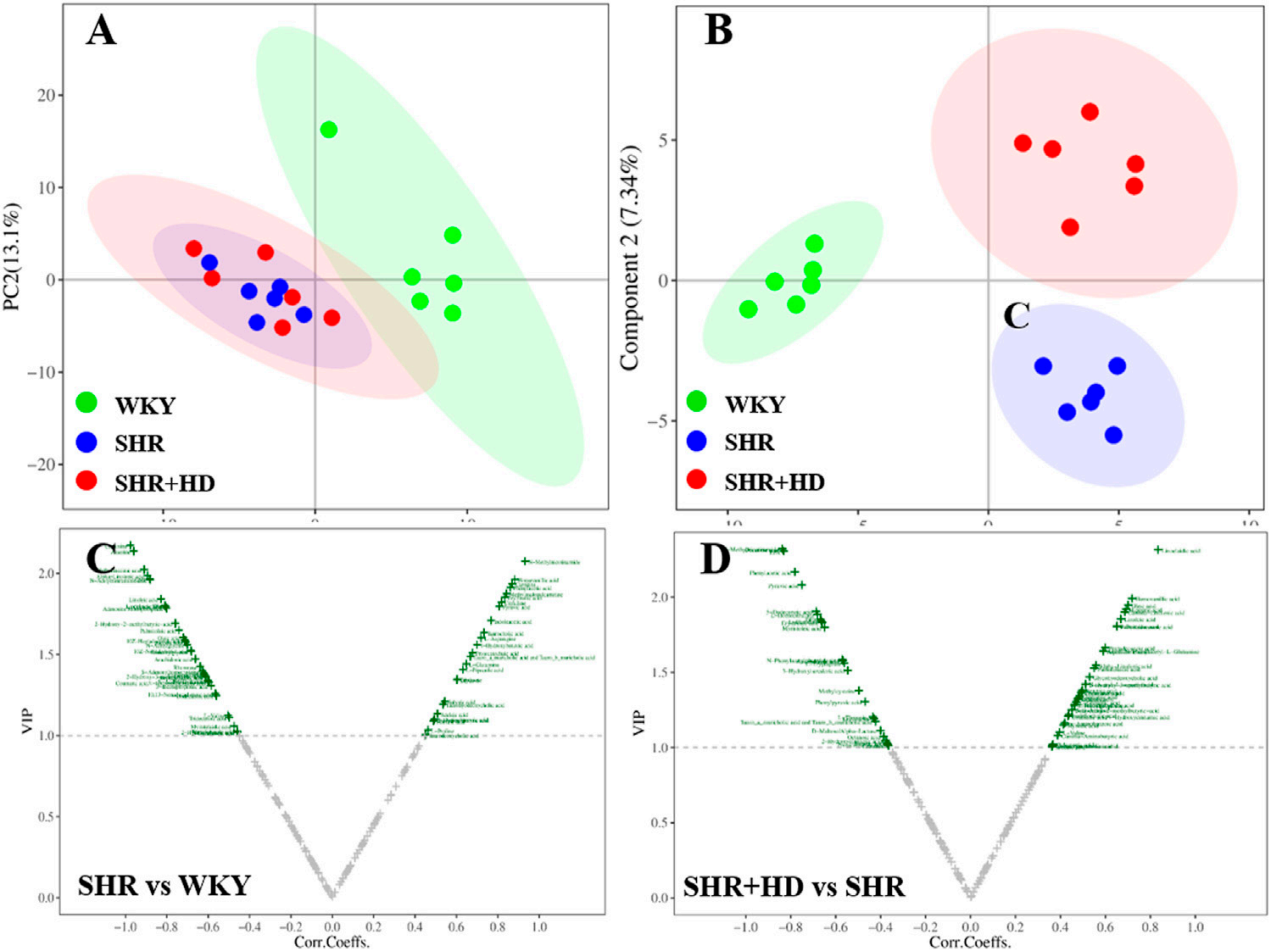
Multivariate statistical analysis of myocardial tissue samples among WKY, SHR, and QSYQ high dose treatment groups. **(A)**, PCA score plot. **(B)**, PLS-DA score plot. **(C,D)**, VIP volcanic map of OPLS-DA model. The PCA and PLS-DA were performed on all groups while the OPLS-DA was performed between two groups. PCA, principal component analysis, PLS-DA, partial least squares discriminant analysis, OPLS-DA, orthogonal projection to the latent structure with discriminant analysis.

In the PCA score plot (Figures 8, 9), the serum and myocardial tissue samples among three groups were tightly aggregated, indicating the stability of the method. Meanwhile, as indicated in the scatter plots of PLS-DA analysis, the plots of the three groups were clearly distinguished (Figures 8, 9) and the global metabolic profiles seemed to reach a new balance after administration of QSYQ.

#### 3.6.1 Identification of the endogenous metabolites and the regulatory effects of QSYQ

To further screen the key metabolites responsible for classification among groups, the OPLS-DA model was applied (Figures 8, 9). According to the screening criteria (VIP >1, *p* < 0.05 and |log_2_FC|>0), totally 23 metabolites in serum samples and myocardial tissue samples could be significantly altered in SHR group compared with WKY group, while QSYQ dramatically call-backed the expression of these common metabolites to normal levels to different extents (Table 2). These metabolites which were regarded as potential biomarkers were mainly associated to fatty acids, carnitines, amino acids, organic acids and other types and indicated a potential mechanism of QSYQ in regulating the cardiac structure and function.

**TABLE 2:** Potential differentiated biomarkers screened from serum and myocardium samples of rats.

Samples	Class	Metabolite	SHR vs. WKY			SHR + HD vs. SHR		
			P	OPLSDA_VIP	Changes	P	OPLSDA_VIP	Changes
Serum	Amino Acids	2-Phenylglycine	3.50E-04	1.59	up	3.22E-03	1.65	down
	Carnitines	3-Hydroxybutyrylcarnitine	1.24E-03	1.4	up	2.16E-03	1.8	down
	Fatty Acids	3-Hydroxyisovaleric acid	7.73E-04	1.48	up	5.36E-06	1.88	down
	Benzenoids	Benzenebutanoic acid	2.16E-03	1.37	up	2.16E-03	1.45	down
	SCFAs	Isovaleric acid	1.52E-02	1.19	up	8.66E-03	1.61	down
	Carnitines	Isovelarylcarnitine	2.92E-03	1.47	up	2.00E-02	1.32	down
	Carnitines	L-Acetylcarnitine	9.43E-04	1.44	up	5.41E-03	1.62	down
	Amino Acids	L-Asparagine	6.24E-03	1.28	up	5.34E-03	1.51	down
	Amino Acids	L-Pipecolic acid	1.10E-02	1.24	up	4.33E-03	1.58	down
	Pyridines	N-Methylnicotinamide	2.16E-03	1.59	up	2.16E-03	1.63	down
	Carnitines	Oleylcarnitine C18 1	4.52E-04	1.5	up	3.98E-02	1.22	down
	Benzenoids	Phenylacetic acid	2.16E-03	1.59	up	2.16E-03	1.65	down
	Nucleosides	S-Adenosylhomocysteine	8.90E-03	1.34	up	1.32E-02	1.48	down
	Carnitines	Stearylcarnitine	3.52E-04	1.51	up	4.11E-02	1.23	down
	Bile Acids	Taurochenodeoxycholic acid	2.16E-03	1.21	up	1.52E-02	1.17	down
	Carbohydrates	Threonic acid	1.67E-03	1.52	up	3.97E-02	1.29	down
myocardium	Organic Acids	Erythronic acid	8.37E-04	1.85	up	1.55E-02	1.83	down
	Fatty Acids	Linoelaidic acid	2.16E-03	1.8	down	2.58E-03	2.31	up
	Fatty Acids	Linoleic acid	4.33E-03	1.84	down	4.11E-02	1.85	up
	Pyridines	N-Methylnicotinamide	2.00E-05	2.07	up	1.27E-03	2.32	down
	Fatty Acids	Oleic acid	8.16E-03	1.6	down	1.53E-02	1.94	up
	Fatty Acids	Palmitoleic acid	9.78E-03	1.64	down	3.34E-02	1.8	up
	Benzenoids	Phenylacetic acid	4.59E-04	1.91	up	3.55E-03	2.16	down
	Organic Acids	Pyruvic acid	3.57E-03	1.79	up	1.00E-02	2.08	down

#### 3.6.2 Identification of the metabolomics pathways of the differential metabolites

**FIGURE 10:**
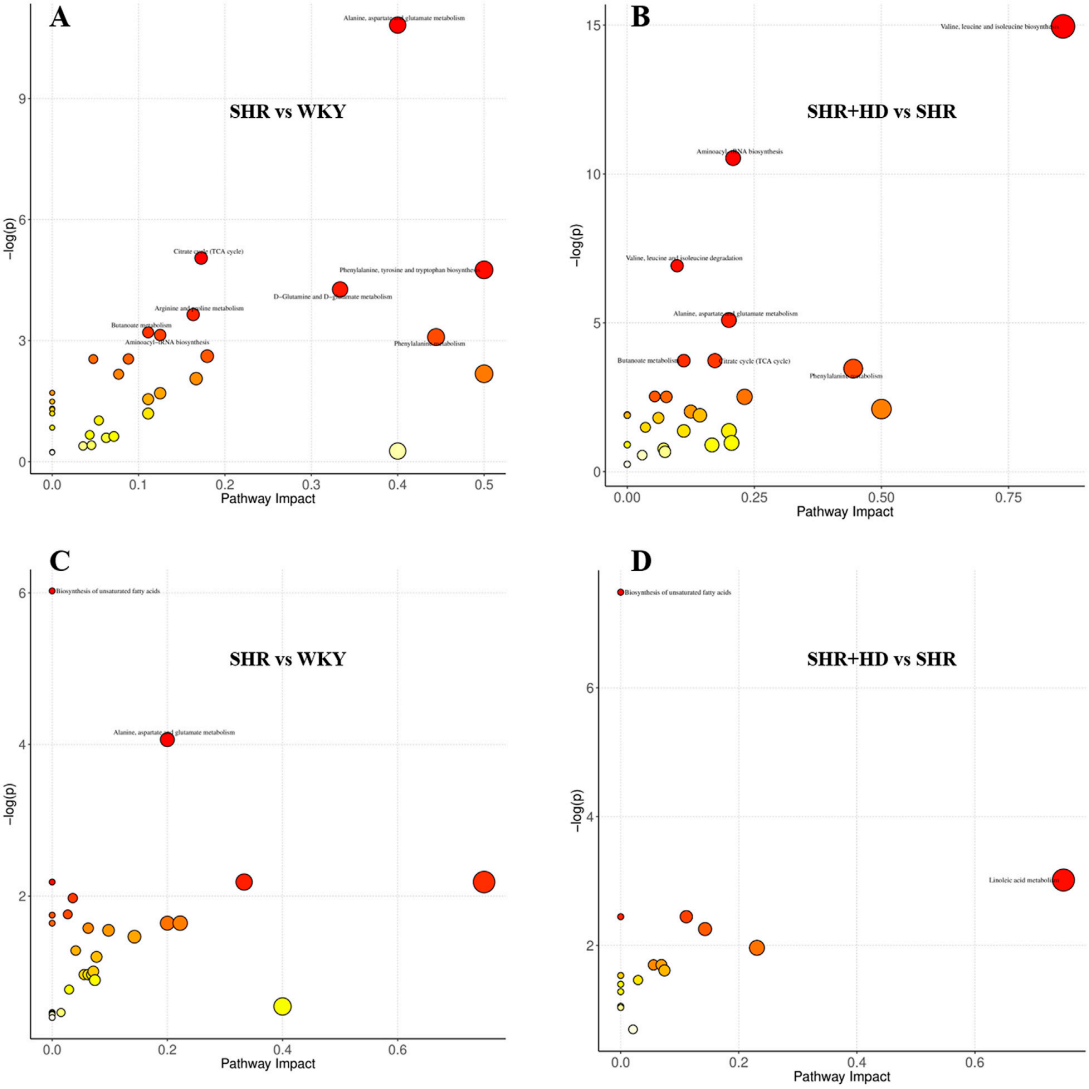
The overview of metabolic pathways analysis based the differential metabolites among WKY, SHR and QSYQ high dose treatment groups. **(A,B)**, from serum samples. **(C,D)**, from myocardial tissue samples.

Metabolic pathway analysis was performed by importing these differential metabolites into MetaboAnalyst (http://www.metaboanalyst.ca/) to further explore the possible mechanisms through which QSYQ regulates the cardiac structure and function. By setting the screening threshold as raw *p* < 0.05, six key metabolic pathways include alanine, aspartate and glutamate metabolism, aminoacyl-tRNA biosynthesis, phenylalanine metabolism, butanoate metabolism, citrate cycle (TCA cycle), biosynthesis of unsaturated fatty acids were screened out as potential target paths among given groups (Figure 10). Among these pathways, alanine, aspartate and glutamate metabolism was affected by L-Aspartic acid and L-Asparagine, and citrate cycle (TCA cycle) was involved with fumaric acid, L-Malic acid, oxoglutaric acid, succinic acid. Besides, phenylacetic acid and linoleic acid, oleic acid were responsible metabolites nodes in phenylalanine metabolism and biosynthesis of unsaturated fatty acids, respectively.

## 4 Discussion

Hypertension is known to be associated with cardiac structure (e.g., myocardial fibrosis, ventricular remodeling and hypertrophy) as well as functional changes. Sustained pressure setting when left uncontrolled will promote the conversion from compensatory to maladaptive phase, named as pathological hypertrophy. Under the condition, cardiomyocyte growth beyond the capacity of the capillaries to fully supply nutrients and oxygen, leading to cardiac hypoxia, cardiac remodeling and fibrosis, generally predisposing to the development of heart failure (Shimizu and Minamino, 2016). As cardiac hypertrophy plays a central role in cardiac remodeling and is an independent risk factor of cardiac events, it is critically important to clarify this process. In this study, we used non-human primates with HF to evaluate the direct function of QSYQ and rodents to assess the effect on preventing the development of pre-heart failure (Sorrentino, 2019; Di Palo and Barone, 2020).

The current study reported that QSYQ treatment once a day for 12 weeks could ameliorate the cardiac systolic and diastolic function in rhesus monkeys with spontaneous chronic heart failure, which was more obvious in the dose of 100 mg/kg. Moreover, administration of QSYQ at higher dose of 465 mg/kg could significantly lower the blood pressure and improve the ventricular remodeling, collagen deposition and fibrosis in SHR-induced myocardial damaged model, namely pre-HF model. Above-mentioned results were in accordance with previous reports in animal models and clinical trials (Lu et al., 2019; Lin et al., 2020; Guan et al., 2021). Moreover, QSYQ demonstrated regulation to the abnormality of serum and myocardial tissue metabolic profiles through several metabolomics pathways. Hence, QSYQ, a modern Chinese medicine, shows potent effects on preventing the progression of cardiac hypertrophy in rats and HF in non-human primates.

To further elucidate the mechanism of QSYQ on aforementioned effects, we used the strategy of network pharmacology to predict the potential targets and pathways as well as metabolomics to explore biomarkers and pathways. Through network pharmacological analysis, chemical constituents, target proteins and pathway networks of QSYQ were obtained, which also reflect the coordinated regulation characteristics of multi-target and multi-pathway of TCM. The possible pathways contained commonly accepted HIF-1 signaling pathway, FoxO signaling pathway, TNF signaling pathway, PI3K-Akt signaling pathway, which were in accordance with previous papers (Yu et al., 2015; Zhang et al., 2019). Furthermore, according to the characteristics of metabolomics, PLS-DA analysis revealed a different metabolic profile among these groups and 23 significantly altered metabolites were screened through OPLS-DA model. Metabolic pathway analysis unveiled that QSYQ exhibited potential therapeutic effects by affecting six metabolic pathways, which were energy metabolism, oxidative stress or other related paths.

**FIGURE 11:**
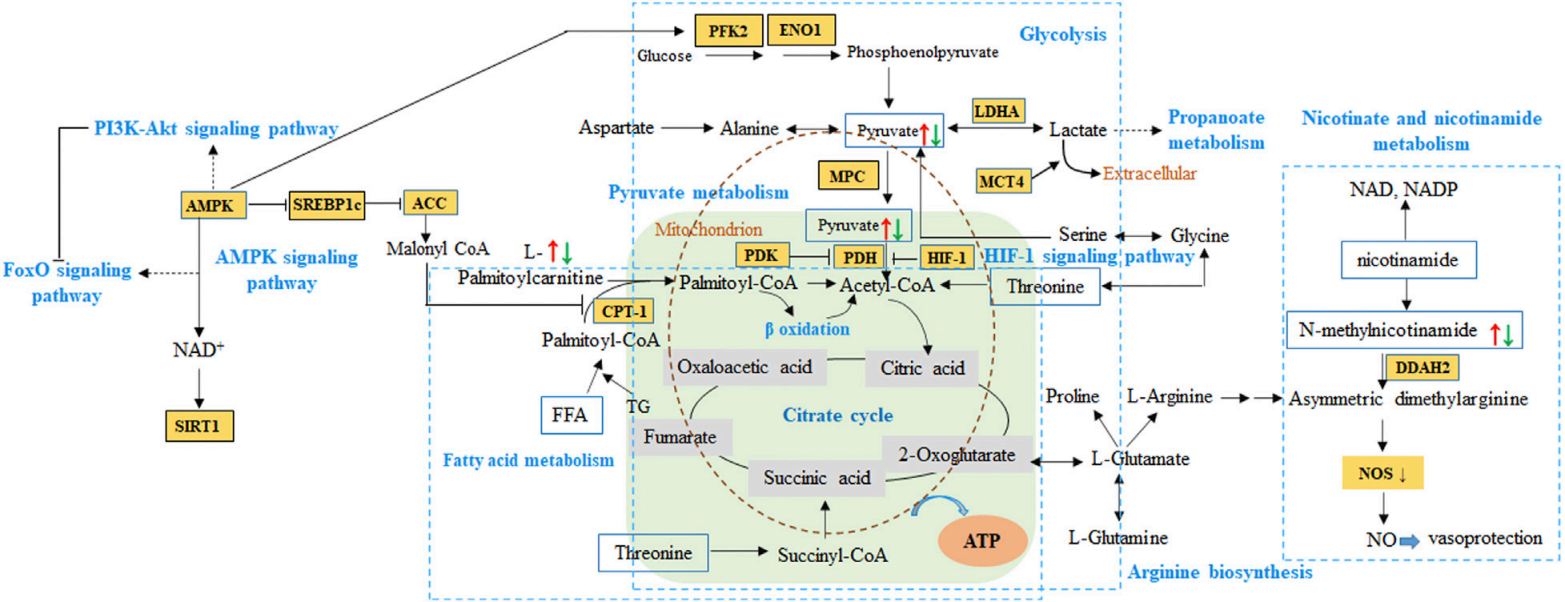
The proposed pathways from network pharmacology and metabolomics integration analysis of QSYQ in HF. Red arrow represented up-regulation of metabolites in model animals, while green referred to down-regulation. HIF-1, hypoxia inducible factor-1α. PDK, pyruvate dehydrogenase kinase. PDH, pyruvate dehydrogenase. LDHA, lactate dehydrogenase. CPT-1, carnitine palmitoyltransferase I. MPC, mitochondrial pyruvate carrier. MCT4, monocarboxylate transporter 4. AMPK, 5′-adenosine monophosphate activated protein kinase. SIRT 1, Sirtuin 1. SREBP1c, sterol regulatory element binding protein-1c. ACC, acetyl-CoA carboxylase. PFK2, phosphor-fructokinase 2. ENO1, enzyme α-enolase. DDAH, dimethylarginine dimethylaminohydrolase. ADMA, asymmetric dimethylarginine.

To our knowledge, ATP is required for cell viability and myocardial pump function, and the balance between fatty acid (FA) β-oxidation and glucose oxidation is an important determinant of cardiac efficiency and function. Under normal conditions, FAs and carbohydrate are the main substrates used by mitochondria to meet the dynamic demands for myocardial energy (Ingwall, 2009). In the cytoplasm of cardiomyocytes (Figure 11), 75% of FA-acyl-coA converted from FAs is transferred into the mitochondria *via* the carnitine palmitoyltransferase (CPT-1 and CPT-2) to undergo β-oxidation. The subsequently produced acetyl-coA enters TCA cycle to produce ATP (Jaswal et al., 2011). In uncompensated hypertrophy and HF, the ventricular remodeling occurs due to the damage to heart and the dynamic relationship between fatty acid β-oxidation and glucose oxidation is perturbed. On the contrary to normal state, the fuel source from fatty acid oxidation gradually switches to glucose uptake and utilization. Therefore, serum level of fatty acids and carnitine increases, 5′-adenosine monophosphate activated protein kinase (AMPK) which decreases the production of malonyl-CoA activates and extend to FoxO signaling pathway as well as PI3K-Akt signaling pathway (Jaswal et al., 2011). It is worth mentioning that malonyl-CoA from acetyl-coA catalyzed by acetyl-CoA carboxylase is deemed as the endogenous inhibitor of CPT-1, thus affects fatty acid oxidation.

As mentioned above during HF, in response to a decrease in the supply of oxygen in the myocardium, an increase of hypoxia inducible factor-1α (HIF-1α) promotes cardiac glycolysis without activation of glucose oxidation (Ikegami et al., 2017). At this point, pyruvate was not transported into the mitochondria by the mitochondrial pyruvate carrier (MPC) and oxidized *via* the pyruvate dehydrogenase (PDH) to continue energy transference. Instead, it is metabolized in the cytosol, primarily reduced to lactate, as well as to other metabolites such as involving in propanoate metabolism (Cluntun et al., 2021). In previous reports on hypertrophic cardiomyopathy patients and mice (Fernandez-Caggiano et al., 2020; Fernandez-Caggiano and Eaton, 2021), decreased MPC expression is sufficient to cause metabolic cardiac remodeling, hypertrophic growth and ultimately heart failure, the abundance determines the extent of pyruvate being metabolically oxidized in the mitochondria. Moreover, it is reported that the monocarboxylate transporter 4 (MCT4) inhibition can prevent and reverse cardiomyocyte hypertrophy, which is consistent with decreased pyruvate flux in mitochondrial (Cluntun et al., 2021). In our present study, metabolomics demonstrated that the expression of serum fatty acids and myocardium pyruvic acid tented to be increase in SHR model and down-regulated after administration, which is consistent with clinical reports. So, the pyruvate-lactate axis as a critical regulatory node for cardiomyocyte growth and function may be a potential regulatory mechanism in QSYQ for HF.

Additionally, as to other types of metabolites such as pyridines, nucleosides play a major role in regulating the cardiovascular function. N-methylnicotinamide, a primary metabolite of nicotinamide, exerts antithrombotic and anti-inflammatory effects through mediating the endothelium function by asymmetric dimethylarginine (ADMA) -dimethylarginine dimethylaminohydrolase 2 (DDAH2) axis and contribute to the vasoprotective properties in mice model (Jiang et al., 2016). In several previous case-control studies, plasma S-adenosylhomocysteine (SAH), other than plasma homocysteine, is reported to be a more sensitive biomarker of cardiovascular disease and might be a better predictor of cardiovascular risks like atherosclerosis formation (Xiao et al., 2015). As screened in our present study, the utilization of metabolites above decreased in model rats, followed by a lower expression after QSYQ administration, which indicated the promising pathways in regulation HF and preventing the development of pre-heart failure.

Therefore, according to the metabolomics analysis, the differentiated metabolites involved in aforementioned metabolic pathways can be mapped to the results predicted by network pharmacology, including HIF-1 signaling pathway, AMPK signaling pathway as well as PI3K-AKT signaling pathway and FoxO signaling pathway. These paths verified by reported studies from experimental and clinical studies tended to be the core metabolic pathway for QSYQ in HF.

According to the theory of traditional Chinese medicine, QSYQ is used as a compound preparation against disease related to “Qi deficiency and blood stasis”. Qi deficiency mainly reflects in the lack of energy to support a variety of biological functions, while blood stasis is caused by disturbance of blood circulation (Zheng et al., 2015)^.^ In this study, QSYQ reinforced Qi efficacy by the regulation of myocardial energy metabolism related pathways, and improved the blood stasis *via* atherosclerosis intervention and promoted blood circulation by nicotinate and nicotinamide metabolism or SAH regulation.

## 5 Conclusion

In summary, as evidenced by pharmacology experiments in non-human primates and rodents, our study showed beneficial effects of QSYQ on parameters related to HF. A systematic exploration of mechanism based on network pharmacology and metabolomics integration analysis received HIF-1 signaling pathway, AMPK signaling pathway, PI3K-AKT signaling pathway, FoxO signaling pathway to potentially regulate state of Qi deficiency and blood stasis in patients. Our study and data support the theory of the underlying mechanism of QSYQ in preventing the development of heart failure, hence lays a foundation on clinical application.

## Data Availability

The original contributions presented in the study are included in the article/Supplementary Material, further inquiries can be directed to the corresponding authors.

## References

[B1] Chang M. Cheng L. Shen Y. Zhang Y. Zhang Z. Hao P. (2019). Qishenyiqi dripping pill improves ventricular remodeling and function in patients with chronic heart failure: A pooled analysis. Med. Baltim. 98 (2), e13906. 10.1097/MD.0000000000013906 PMC6336621 30633164

[B2] Chen L. Wang R. Liu H. Wei S. Jing M. Wang M. (2021). Clinical efficacy and safety of qishen yiqi dropping pill combined with conventional western medicine in the treatment of chronic heart failure: A systematic review and meta-analysis. Evid. Based. Complement. Altern. Med. 2021, 6612653. 10.1155/2021/6612653 PMC7872761 33603818

[B3] Cluntun A. A. Badolia R. Lettlova S. Parnell K. M. Shankar T. S. Diakos N. A. (2021). The pyruvate-lactate axis modulates cardiac hypertrophy and heart failure. Cell. Metab. 33 (3), 629–648.e10. 10.1016/j.cmet.2020.12.003 33333007 PMC7933116

[B4] Di Palo K. E. Barone N. J. (2020). Hypertension and heart failure: Prevention, targets, and treatment. Heart fail. Clin. 16 (1), 99–106. 10.1016/j.hfc.2019.09.001 31735319

[B5] Fernandez-Caggiano M. Eaton P. (2021). Heart failure-emerging roles for the mitochondrial pyruvate carrier. Cell. Death Differ. 28 (4), 1149–1158. 10.1038/s41418-020-00729-0 33473180 PMC8027425

[B6] Fernandez-Caggiano M. Kamynina A. Francois A. A. Prysyazhna O. Eykyn T. R. Krasemann S. (2020). Mitochondrial pyruvate carrier abundance mediates pathological cardiac hypertrophy. Nat. Metab. 2 (11), 1223–1231. 10.1038/s42255-020-00276-5 33106688 PMC7610404

[B7] Guan H. Dai G. Ren L. Gao W. Fu H. Zhao Z. (2021). Efficacy and safety of qishen yiqi dripping pills as a complementary treatment for heart failure: A protocol of updated systematic review and meta-analysis. Med. Baltim. 100 (2), e24285. 10.1097/MD.0000000000024285 PMC7808537 33466215

[B8] Hamosh A. Scott A. F. Amberger J. S. Bocchini C. A. McKusick V. A. (2005). Online Mendelian Inheritance in Man (OMIM), a knowledgebase of human genes and genetic disorders. Nucleic Acids Res. 33, D514–D517. 10.1093/nar/gki033 15608251 PMC539987

[B9] Holscher M. Schafer K. Krull S. Farhat K. Hesse A. Silter M. (2012). Unfavourable consequences of chronic cardiac HIF-1α stabilization. Cardiovasc. Res. 94 (1), 77–86. 10.1093/cvr/cvs014 22258630

[B10] Hong L. Y. Yan Y. C. Xu L. X. Peng Z. Jing T. Yang Q. (2017). Therapeutic target database update 2018: Enriched resource for facilitating bench-to-clinic research of targeted therapeutics. Nucleic Acids Res. 46, D1121–D1127. 10.1093/nar/gkx1076 PMC5753365 29140520

[B11] Huang da W. Sherman B. T. Lempicki R. A. (2009). Systematic and integrative analysis of large gene lists using DAVID bioinformatics resources. Nat. Protoc. 4 (1), 44–57. 10.1038/nprot.2008.211 19131956

[B12] Ikegami R. Shimizu I. Yoshida Y. Minamino T. (2017). Metabolomic analysis in heart failure. Circ. J. 82 (1), 10–16. 10.1253/circj.CJ-17-1184 29225300

[B13] Ingwall J. S. (2009). Energy metabolism in heart failure and remodelling. Cardiovasc. Res. 81 (3), 412–419. 10.1093/cvr/cvn301 18987051 PMC2639129

[B14] Janet P. lex B. Núria Q. R. Alba G. S. Jordi D. P. Emilio C. (2016). DisGeNET: A comprehensive platform integrating information on human disease-associated genes and variants. Nucleic Acids Res. 45, D833–D839. 10.1093/nar/gkw943 27924018 PMC5210640

[B15] Jaswal J. S. Keung W. Wang W. Ussher J. R. Lopaschuk G. D. (2011). Targeting fatty acid and carbohydrate oxidation--a novel therapeutic intervention in the ischemic and failing heart. Biochim. Biophys. Acta 1813 (7), 1333–1350. 10.1016/j.bbamcr.2011.01.015 21256164

[B16] Jiang N. Wang M. Song J. Liu Y. Chen H. Mu D. (2016). N-methylnicotinamide protects against endothelial dysfunction and attenuates atherogenesis in apolipoprotein E-deficient mice. Mol. Nutr. Food Res. 60 (7), 1625–1636. 10.1002/mnfr.201501019 26887666

[B17] Lin S. S. Liu C. X. Zhang J. H. Wang X. L. Mao J. Y. (2020). Efficacy and safety of oral Chinese patent medicine combined with conventional therapy for heart failure: An overview of systematic reviews. Evid. Based. Complement. Altern. Med. 2020, 8620186. 10.1155/2020/8620186 PMC7474350 32908572

[B18] Liu Y. Wang Y. Ni Y. Cheung C. K. Y. Lam K. S. L. Wang Y. (2020). Gut microbiome fermentation determines the efficacy of exercise for diabetes prevention. Cell. Metab. 31 (1), 77–91. 10.1016/j.cmet.2019.11.001 31786155

[B19] Lu Y. Wang D. Yuan X. Wang M. Li Z. Bao X. (2019). Protective effect of Qishen Yiqi dropping pills on the myocardium of rats with chronic heart failure. Exp. Ther. Med. 17 (1), 378–382. 10.3892/etm.2018.6957 30651807 PMC6307364

[B20] Luo T. T. Lu Y. Yan S. K. Xiao X. Rong X. L. Guo J. (2020). Network pharmacology in research of Chinese medicine formula: Methodology, application and prospective. Chin. J. Integr. Med. 26 (1), 72–80. 10.1007/s11655-019-3064-0 30941682

[B21] Nagueh S. F. Middleton K. J. Kopelen H. A. Zoghbi W. A. Quinones M. A. (1997). Doppler tissue imaging: A noninvasive technique for evaluation of left ventricular relaxation and estimation of filling pressures. J. Am. Coll. Cardiol. 30 (6), 1527–1533. 10.1016/s0735-1097(97)00344-6 9362412

[B22] Ru J. Li P. Wang J. Zhou W. Li B. Huang C. (2014). Tcmsp: A database of systems pharmacology for drug discovery from herbal medicines. J. Cheminform. 6, 13. 10.1186/1758-2946-6-13 24735618 PMC4001360

[B23] Shao Y. Zhang W. Tong L. Huang J. Li D. Nie W. (2017). Simultaneous determination of eight bioactive components of Qishen Yiqi dripping pills in rat plasma using UFLC-MS/MS and its application to a pharmacokinetic study. Biomed. Chromatogr. 31 (8), e3941. 10.1002/bmc.3941 28146302

[B24] Shen M. Tian S. Li Y. Li Q. Xu X. Wang J. (2012). Drug-likeness analysis of traditional Chinese medicines: 1. Property distributions of drug-like compounds, non-drug-like compounds and natural compounds from traditional Chinese medicines. J. Cheminform. 4 (1), 31. 10.1186/1758-2946-4-31 23181938 PMC3538521

[B25] Shimizu I. Minamino T. (2016). Physiological and pathological cardiac hypertrophy. J. Mol. Cell. Cardiol. 97, 245–262. 10.1016/j.yjmcc.2016.06.001 27262674

[B26] Sorrentino M. J. (2019). The evolution from hypertension to heart failure. Heart fail. Clin. 15 (4), 447–453. 10.1016/j.hfc.2019.06.005 31472880

[B27] Su X. Yao Z. Li S. Sun H. (2016). Synergism of Chinese herbal medicine: Illustrated by danshen compound. Evid. Based. Complement. Altern. Med. 2016, 7279361. 10.1155/2016/7279361 PMC4846759 27190537

[B28] Tanai E. Frantz S. (2015). Pathophysiology of heart failure. Compr. Physiol. 6 (1), 187–214. 10.1002/cphy.c140055 26756631

[B29] Tham Y. K. Bernardo B. C. Ooi J. Y. Weeks K. L. McMullen J. R. (2015). Pathophysiology of cardiac hypertrophy and heart failure: Signaling pathways and novel therapeutic targets. Arch. Toxicol. 89 (9), 1401–1438. 10.1007/s00204-015-1477-x 25708889

[B30] Whirl‐Carrillo M. Mcdonagh E. M. Hebert J. M. Gong L. Sangkuhl K. Thorn C. F. (2012). Pharmacogenomics knowledge for personalized medicine. Clin. Pharmacol. Ther. 92, 414–417. 10.1038/clpt.2012.96 22992668 PMC3660037

[B31] Wishart D. S. Feunang Y. D. Guo A. C. Lo E. J. Marcu A. Grant J. R. (2018). DrugBank 5.0: A major update to the DrugBank database for 2018. Nucleic Acids Res. 46 (D1), D1074–D1082. 10.1093/nar/gkx1037 29126136 PMC5753335

[B32] Xiao Y. Huang W. Zhang J. Peng C. Xia M. Ling W. (2015). Increased plasma S-adenosylhomocysteine-accelerated atherosclerosis is associated with epigenetic regulation of endoplasmic reticulum stress in apoE-/- mice. Arterioscler. Thromb. Vasc. Biol. 35 (1), 60–70. 10.1161/ATVBAHA.114.303817 25359864

[B33] Xin Z. Ma Z. Hu W. Jiang S. Yang Z. Li T. (2018). FOXO1/3: Potential suppressors of fibrosis. Ageing Res. Rev. 41, 42–52. 10.1016/j.arr.2017.11.002 29138094

[B34] Xu X. Zhang W. Huang C. Li Y. Yu H. Wang Y. (2012). A novel chemometric method for the prediction of human oral bioavailability. Int. J. Mol. Sci. 13 (6), 6964–6982. 10.3390/ijms13066964 22837674 PMC3397506

[B35] Yu F.-C. Xu Y.-J. Tong J.-Y. Lu Z.-Z. Zhang X.-H. (2015). Therapeutic effects of Qishen Yiqi Dropping Pill on myocardial injury induced by chronic hypoxia in rats. Chin. J. Nat. Med. 13 (10), 776–780. 10.1016/s1875-5364(15)30078-9 26481378

[B36] Yunfei L. Haibin Q. Yiyu C. (2008). Identification of major constituents in the traditional Chinese medicine "QI-SHEN-YI-QI" dropping pill by high-performance liquid chromatography coupled with diode array detection-electrospray ionization tandem mass spectrometry. J. Pharm. Biomed. Anal. 47 (2), 407–412. 10.1016/j.jpba.2007.12.037 18387767

[B37] Zhang S. Wang H. Li L. Chang X. Ma H. Zhang M. (2019). Qishen Yiqi Drop Pill, a novel compound Chinese traditional medicine protects against high glucose-induced injury in cardiomyocytes. J. Cell. Mol. Med. 23 (9), 6393–6402. 10.1111/jcmm.14527 31278860 PMC6714141

[B38] Zhang Y. Shi P. Yao H. Shao Q. Fan X. (2012). Metabolite profiling and pharmacokinetics of herbal compounds following oral administration of a cardiovascular multi-herb medicine (Qishen yiqi pills) in rats. Curr. Drug Metab. 13 (5), 510–523. 10.2174/1389200211209050510 22292791

[B39] Zhao C. Wang W. Yan K. Sun H. Han J. Hu Y. (2021). The therapeutic effect and mechanism of Qishen Yiqi dripping pills on cardiovascular and cerebrovascular diseases and diabetic complications. Curr. Mol. Pharmacol. 15, 547–556. 10.2174/1874467214666210811153610 34382512

[B40] Zheng S. Zhang Y. Qiao Y. (2015). The mechanism research of qishen yiqi formula by module-network analysis. Evid. Based. Complement. Altern. Med. 2015, 497314. 10.1155/2015/497314 PMC4561322 26379745

