# Corrigendum: Integrated network pharmacology and metabolomics to reveal the mechanism of QiShenYiQi Dripping Pills against cardiac structural and functional abnormalities

**DOI:** 10.3389/fphar.2022.1080645

**Published:** 2022-11-21

**Authors:** Jun Zhang, Zunyuan Yang, Xue Jia, Xinxin Li, Xiangyang Wang, Hua Rong, Yinan Liang, Wen Zeng, Wei Jia, Xiaohui Ma

**Affiliations:** ^1^ The State Key Laboratory of Core Technology in Innovative Chinese Medicine, Tasly Academy, Tasly Holding Group Co., Ltd, Tianjin, China; ^2^ PriMed Non-Human Primate Research Center of Sichuan PriMed Shines Bio-Tech Co., Ltd., Ya’an, Sichuan, China; ^3^ China Pharmaceutical University, Nanjing, China; ^4^ Center for Translational Medicine and Shanghai Key Laboratory of Diabetes Mellitus, Shanghai Jiao Tong University Affiliated Sixth People’s Hospital, Shanghai, China; ^5^ School of Chinese Medicine, Hong Kong Baptist University, Hong Kong, China

**Keywords:** heart failure, QSYQ, SHR, network pharmacology, metabolomics

In the original article, there was an error in the name of Traditional Chinese Medicine throughout the article. The name was displayed as “QiShenYiQi Dripping Pills (T101)” or “QSYQ (T101)”. The corrected name is “QiShenYiQi Dripping Pills” or “QSYQ”. The code number T101 has been deleted in the **title** and Figure 1.

**FIGURE 1:**
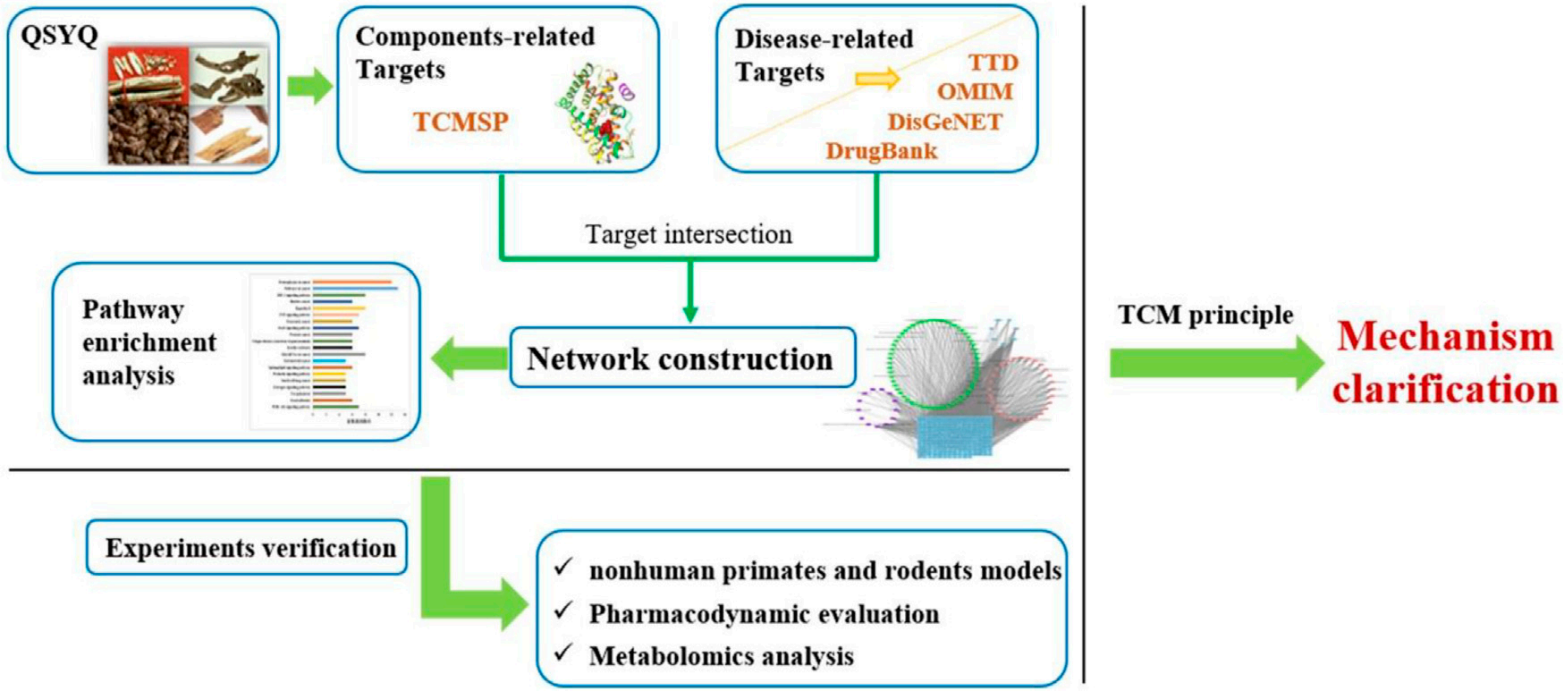
The flowchart of the study.

The corrected **title** appears below.

“Integrated network pharmacology and metabolomics to reveal the mechanism of QiShenYiQi Dripping Pills against cardiac structural and functional abnormalities”.

The corrected Figure 1 and its caption “The flowchart of the study.” appear below.

The authors apologize for this error and state that this does not change the scientific conclusions of the article in any way. The original article has been updated.

