# Supplementary material for: Integrated network pharmacology and metabolomics to reveal the mechanism of QiShenYiQi Dripping Pills against cardiac structural and functional abnormalities
Source: Front Pharmacol. 2022 Oct 24;13:1017433. doi: 10.3389/fphar.2022.1017433 (PMC9638142; doi:10.3389/fphar.2022.1017433)
Supplement: Supplementary file 1 [file DataSheet1.docx]

Supplementary Material

# Methods and Materials

# 1.1 Targeted Metabolomics Profiling

Sample preparation and instrument parameter setting can be referred to published studies (Liu et al., 2020). Briefly, samples were thawed on ice-bath to diminish sample degradation. For myocardial tissue samples, each tissue sample (~10mg) that was harvested and stored in an Eppendorf Safelock microcentrifuge tube, was mixed with 10 pre-chilled zirconium oxide beads and 20 μL of deionized water. The tissue sample was homogenated for 3 minutes and 100~150 μL of ice-cold methanol containing internal standard was added into above-mentioned homogenate or 25 μL of serum sample to extract the metabolites. Then the plate with supernatant was sealed and the derivatization was carried out at 30°C for 60 min. After derivatization, 50% methanol solution was added to dilute the sample to get supernatant. Finally, the plate was sealed for LC-MS analysis. An ultra-performance liquid chromatography coupled to tandem mass spectrometry (UPLC-MS/MS) system (ACQUITY UPLC-Xevo TQ-S, Waters Corp., Milford, MA, USA) was used to quantitate functional small molecule metabolites. All of the standards of targeted metabolites were obtained from Sigma-Aldrich (St. Louis, MO, USA), Steraloids Inc. (Newport, RI, USA) and TRC Chemicals (Toronto, ON, Canada).

The raw data files generated by UPLC-MS/MS were processed using the iMAP software (v1.0.0, Metabo-Profile, Shanghai, China) to perform peak integration, calibration, and quantitation for each metabolite. By comparing the unknown to a set of standard samples of known concentration (i.e., calibration curve), the concentration of a substance in an unknown sample could be determined.

As to statistical analysis, both principal component analysis (PCA) and orthogonal projection to latent structures square-discriminate analysis (OPLS-DA) were performed. The V-plot of the OPLS-DA model was used to visualize metabolites, while the candidate biomarkers were screened through multivariate and univariate statistical analysis by Variable importance in projection (VIP) > 1, *P* < 0.05 and |log_2_FC|>0 (Fold Change). Moreover, metabolic pathway analysis was performed by MetaboAnalyst 4.0 (https://www.metaboanalyst.ca/) to reveal disturbed metabolism. Pathways with the values of *P* < 0.05 were screened out as the candidate target pathways.

# Results

## Active ingredients and targets screened from TCMSP

Traditional Chinese Medicine Systems Pharmacology Database (TCMSP, http://tcmspw.com/tcmspsearch.php) was used to retrieve the compound information (Supplementary Table 1) of QSYQ (T101) and the related targets (Supplementary Table 2). The screening criteria for components were set as the oral bioavailability (OB) greater than 30% and the drug likeness (DL) greater than 0.18.

**Supplementary Table 1** Potential ingredients details of herbs

| **Herb** | **Mol ID** | **Ingredients** | **OB(%)** | **DL** |
| --- | --- | --- | --- | --- |
| Hedysarum Multijugum Maxim.  (20) | MOL000211 | Mairin | 55.38 | 0.78 |
|  | MOL000239 | Jaranol | 50.83 | 0.29 |
|  | MOL000296 | hederagenin | 36.91 | 0.75 |
|  | MOL000033 | (3S,8S,9S,10R,13R,14S,17R)-10,13-dimethyl-17-[(2R,5S)-5-propan-2-yloctan-2-yl]-2,3,4,7,8,9,11,12,14,15,16,17-dodecahydro-1H-cyclopenta[a]phenanthren-3-ol | 36.23 | 0.78 |
|  | MOL000354 | isorhamnetin | 49.6 | 0.31 |
|  | MOL000371 | 3,9-di-O-methylnissolin | 53.74 | 0.48 |
|  | MOL000374 | 5'-hydroxyiso-muronulatol-2',5'-di-O-glucoside | 41.72 | 0.69 |
|  | MOL000378 | 7-O-methylisomucronulatol | 74.69 | 0.3 |
|  | MOL000379 | 9,10-dimethoxypterocarpan-3-O-β-D-glucoside | 36.74 | 0.92 |
|  | MOL000380 | (6aR,11aR)-9,10-dimethoxy-6a,11a-dihydro-6H-benzofurano[3,2-c]chromen-3-ol | 64.26 | 0.42 |
|  | MOL000387 | Bifendate | 31.1 | 0.67 |
|  | MOL000392 | formononetin | 69.67 | 0.21 |
|  | MOL000398 | isoflavanone | 109.99 | 0.3 |
|  | MOL000417 | Calycosin | 47.75 | 0.24 |
|  | MOL000422 | kaempferol | 41.88 | 0.24 |
|  | MOL000433 | FA | 68.96 | 0.71 |
|  | MOL000438 | (3R)-3-(2-hydroxy-3,4-dimethoxyphenyl)chroman-7-ol | 67.67 | 0.26 |
|  | MOL000439 | isomucronulatol-7,2'-di-O-glucosiole | 49.28 | 0.62 |
|  | MOL000442 | 1,7-Dihydroxy-3,9-dimethoxy pterocarpene | 39.05 | 0.48 |
|  | MOL000098 | quercetin | 46.43 | 0.28 |
| Salvia miltiorrhiza  (67) | MOL001601 | 1,2,5,6-tetrahydrotanshinone | 38.75 | 0.36 |
|  | MOL001659 | Poriferasterol | 43.83 | 0.76 |
|  | MOL001771 | poriferast-5-en-3beta-ol | 36.91 | 0.75 |
|  | MOL001942 | isoimperatorin | 45.46 | 0.23 |
|  | MOL002222 | sugiol | 36.11 | 0.28 |
|  | MOL002651 | Dehydrotanshinone II A | 43.76 | 0.4 |
|  | MOL002776 | Baicalin | 40.12 | 0.75 |
|  | MOL000569 | digallate | 61.85 | 0.26 |
|  | MOL000006 | luteolin | 36.16 | 0.25 |
|  | MOL006824 | α-amyrin | 39.51 | 0.76 |
|  | MOL007036 | 5,6-dihydroxy-7-isopropyl-1,1-dimethyl-2,3-dihydrophenanthren-4-one | 33.77 | 0.29 |
|  | MOL007041 | 2-isopropyl-8-methylphenanthrene-3,4-dione | 40.86 | 0.23 |
|  | MOL007045 | 3α-hydroxytanshinoneⅡa | 44.93 | 0.44 |
|  | MOL007048 | (E)-3-[2-(3,4-dihydroxyphenyl)-7-hydroxy-benzofuran-4-yl]acrylic acid | 48.24 | 0.31 |
|  | MOL007049 | 4-methylenemiltirone | 34.35 | 0.23 |
|  | MOL007050 | 2-(4-hydroxy-3-methoxyphenyl)-5-(3-hydroxypropyl)-7-methoxy-3-benzofurancarboxaldehyde | 62.78 | 0.4 |
|  | MOL007051 | 6-o-syringyl-8-o-acetyl shanzhiside methyl ester | 46.69 | 0.71 |
|  | MOL007058 | formyltanshinone | 73.44 | 0.42 |
|  | MOL007059 | 3-beta-Hydroxymethyllenetanshiquinone | 32.16 | 0.41 |
|  | MOL007061 | Methylenetanshinquinone | 37.07 | 0.36 |
|  | MOL007063 | przewalskin a | 37.11 | 0.65 |
|  | MOL007064 | przewalskin b | 110.32 | 0.44 |
|  | MOL007068 | Przewaquinone B | 62.24 | 0.41 |
|  | MOL007069 | przewaquinone c | 55.74 | 0.4 |
|  | MOL007070 | (6S,7R)-6,7-dihydroxy-1,6-dimethyl-8,9-dihydro-7H-naphtho[8,7-g]benzofuran-10,11-dione | 41.31 | 0.45 |
|  | MOL007071 | przewaquinone f | 40.31 | 0.46 |
|  | MOL007077 | sclareol | 43.67 | 0.21 |
|  | MOL007079 | tanshinaldehyde | 52.47 | 0.45 |
|  | MOL007081 | Danshenol B | 57.95 | 0.56 |
|  | MOL007082 | Danshenol A | 56.97 | 0.52 |
|  | MOL007085 | Salvilenone | 30.38 | 0.38 |
|  | MOL007088 | cryptotanshinone | 52.34 | 0.4 |
|  | MOL007093 | dan-shexinkum d | 38.88 | 0.55 |
|  | MOL007094 | danshenspiroketallactone | 50.43 | 0.31 |
|  | MOL007098 | deoxyneocryptotanshinone | 49.4 | 0.29 |
|  | MOL007100 | dihydrotanshinlactone | 38.68 | 0.32 |
|  | MOL007101 | dihydrotanshinoneⅠ | 45.04 | 0.36 |
|  | MOL007105 | epidanshenspiroketallactone | 68.27 | 0.31 |
|  | MOL007107 | C09092 | 36.07 | 0.25 |
|  | MOL007108 | isocryptotanshi-none | 54.98 | 0.39 |
|  | MOL007111 | Isotanshinone II | 49.92 | 0.4 |
|  | MOL007115 | manool | 45.04 | 0.2 |
|  | MOL007118 | microstegiol | 39.61 | 0.28 |
|  | MOL007119 | miltionone Ⅰ | 49.68 | 0.32 |
|  | MOL007120 | miltionone Ⅱ | 71.03 | 0.44 |
|  | MOL007121 | miltipolone | 36.56 | 0.37 |
|  | MOL007122 | Miltirone | 38.76 | 0.25 |
|  | MOL007123 | miltirone Ⅱ | 44.95 | 0.24 |
|  | MOL007124 | neocryptotanshinone ii | 39.46 | 0.23 |
|  | MOL007125 | neocryptotanshinone | 52.49 | 0.32 |
|  | MOL007127 | 1-methyl-8,9-dihydro-7H-naphtho[5,6-g]benzofuran-6,10,11-trione | 34.72 | 0.37 |
|  | MOL007130 | prolithospermic acid | 64.37 | 0.31 |
|  | MOL007132 | (2R)-3-(3,4-dihydroxyphenyl)-2-[(Z)-3-(3,4-dihydroxyphenyl)acryloyl]oxy-propionic acid | 109.38 | 0.35 |
|  | MOL007140 | (Z)-3-[2-[(E)-2-(3,4-dihydroxyphenyl)vinyl]-3,4-dihydroxy-phenyl]acrylic acid | 88.54 | 0.26 |
|  | MOL007141 | salvianolic acid g | 45.56 | 0.61 |
|  | MOL007142 | salvianolic acid j | 43.38 | 0.72 |
|  | MOL007143 | salvilenone Ⅰ | 32.43 | 0.23 |
|  | MOL007145 | salviolone | 31.72 | 0.24 |
|  | MOL007149 | NSC 122421 | 34.49 | 0.28 |
|  | MOL007150 | (6S)-6-hydroxy-1-methyl-6-methylol-8,9-dihydro-7H-naphtho[8,7-g]benzofuran-10,11-quinone | 75.39 | 0.46 |
|  | MOL007151 | Tanshindiol B | 42.67 | 0.45 |
|  | MOL007152 | Przewaquinone E | 42.85 | 0.45 |
|  | MOL007154 | tanshinone iia | 49.89 | 0.4 |
|  | MOL007155 | (6S)-6-(hydroxymethyl)-1,6-dimethyl-8,9-dihydro-7H-naphtho[8,7-g]benzofuran-10,11-dione | 65.26 | 0.45 |
|  | MOL007156 | tanshinone Ⅵ | 45.64 | 0.3 |
|  | --- | Danshensu | --- | --- |
|  | --- | protocatechuic aldehyde | --- | --- |
| Panax Notoginseng  (8) | MOL001494 | Mandenol | 42 | 0.19 |
|  | MOL001792 | DFV | 32.76 | 0.18 |
|  | MOL002879 | Diop | 43.59 | 0.39 |
|  | MOL000358 | beta-sitosterol | 36.91 | 0.75 |
|  | MOL000449 | Stigmasterol | 43.83 | 0.76 |
|  | MOL005344 | ginsenoside rh2 | 36.32 | 0.56 |
|  | MOL007475 | ginsenoside f2 | 36.43 | 0.25 |
|  | MOL000098 | quercetin | 46.43 | 0.28 |
| Dalbergiae Odoriferae Lignum  (36) | MOL001040 | (2R)-5,7-dihydroxy-2-(4-hydroxyphenyl)chroman-4-one | 42.36 | 0.21 |
|  | MOL001792 | DFV | 32.76 | 0.18 |
|  | MOL000228 | (2R)-7-hydroxy-5-methoxy-2-phenylchroman-4-one | 55.23 | 0.2 |
|  | MOL002565 | Medicarpin | 49.22 | 0.34 |
|  | MOL002914 | Eriodyctiol (flavanone) | 41.35 | 0.24 |
|  | MOL002938 | (3R)-4'-Methoxy-2',3,7-trihydroxyisoflavanone | 68.86 | 0.27 |
|  | MOL002939 | (3R)-5'-Methoxyvestitol | 83.06 | 0.26 |
|  | MOL002940 | (3R)-3-(2,3-dihydroxy-4-methoxyphenyl)-7-hydroxychroman-4-one | 52.06 | 0.27 |
|  | MOL002941 | (3R)-3-(2,3-dihydroxy-4-methoxyphenyl)chroman-7,8-diol | 82.35 | 0.27 |
|  | MOL002950 | (3R)-7,2',3'-trihydroxy-4-methoxyisoflavan | 69.65 | 0.24 |
|  | MOL002957 | 9-O-Methylcoumestrol | 33.73 | 0.38 |
|  | MOL002958 | 3'-Hydroxymelanettin | 30.69 | 0.27 |
|  | MOL002959 | 3'-Methoxydaidzein | 48.57 | 0.24 |
|  | MOL002961 | (-)-Vestitol | 70.29 | 0.21 |
|  | MOL002962 | (3S)-7-hydroxy-3-(2,3,4-trimethoxyphenyl)chroman-4-one | 48.23 | 0.33 |
|  | MOL002963 | 4',5',7-trimethyl-3-methoxyflavone | 40.66 | 0.25 |
|  | MOL002966 | Dalbergin | 78.18 | 0.2 |
|  | MOL002967 | 7-hydroxy-4'-methoxy-2',5'-dioxo-4-[(3R)-2',7-dihydroxy-4'-methoxyisoflavan-5'-yl]isoflavane | 34.78 | 0.7 |
|  | MOL002973 | Bowdichione | 55.78 | 0.28 |
|  | MOL002975 | butin | 69.94 | 0.21 |
|  | MOL002981 | Duartin | 70.63 | 0.34 |
|  | MOL002982 | (3R,4R)-3',7-dihydroxy-2',4'-dimethoxy-4-[(2S)-4',5,7-trihydroxyflavanone-6-yl]isoflavan | 33.96 | 0.63 |
|  | MOL002985 | isoduartin | 74.11 | 0.34 |
|  | MOL002989 | 4-Hydroxyhomopterocarpin | 48.41 | 0.43 |
|  | MOL002990 | (6aR,11aR)-3,9,10-trimethoxy-6a,11a-dihydro-6H-benzofurano[3,2-c]chromen-4-ol | 66.86 | 0.53 |
|  | MOL002991 | (6aR,11aR)-3,9-dimethoxy-6a,11a-dihydro-6H-benzofurano[3,2-c]chromene-4,10-diol | 38.96 | 0.48 |
|  | MOL002996 | odoricarpin | 55.02 | 0.53 |
|  | MOL002997 | 3-(2-hydroxy-3,4-dimethoxyphenyl)-2H-chromen-7-ol | 86.18 | 0.27 |
|  | MOL002999 | Sativanone | 85.63 | 0.27 |
|  | MOL003001 | Vestitone | 52.83 | 0.24 |
|  | MOL003002 | violanone | 80.24 | 0.3 |
|  | MOL003003 | Xenognosin B | 72.71 | 0.24 |
|  | MOL000358 | beta-sitosterol | 36.91 | 0.75 |
|  | MOL000359 | sitosterol | 36.91 | 0.75 |
|  | MOL000380 | (6aR,11aR)-9,10-dimethoxy-6a,11a-dihydro-6H-benzofurano [3,2-c]chromen-3-ol | 64.26 | 0.42 |
|  | MOL000392 | formononetin | 69.67 | 0.21 |

**Supplementary Table 2** Components-related Targets from TCMSP database

| **Target name** | **Gene name** |
| --- | --- |
| 26S proteasome non-ATPase regulatory subunit 3 | PSMD3 |
| 3 beta-hydroxysteroid dehydrogenase/Delta 5-->4-isomerase type 1 | HSD3B1 |
| 3 beta-hydroxysteroid dehydrogenase/Delta 5-->4-isomerase type 2 | HSD3B2 |
| 5-hydroxytryptamine 1A receptor | HTR1A |
| 5-hydroxytryptamine 1B receptor | HTR1B |
| 5-hydroxytryptamine 2A receptor | HTR2A |
| 5-hydroxytryptamine 2C receptor | HTR2C |
| 5-hydroxytryptamine receptor 3A | HTR3A |
| 72 kDa type IV collagenase | MMP2 |
| 78 kDa glucose-regulated protein | HSPA5 |
| Acetylcholinesterase | ACHE |
| Acetyl-CoA carboxylase 1 | ACACA |
| Actin, cytoplasmic 1 | ACTB |
| Activator of 90 kDa heat shock protein ATPase homolog 1 | AHSA1 |
| Adenylate cyclase type 2 | ADCY2 |
| Alcohol dehydrogenase 1B | ADH1B |
| Alcohol dehydrogenase 1C | ADH1C |
| Aldo-keto reductase family 1 member C3 | AKR1C3 |
| Aldose reductase | AKR1B1 |
| Alpha-1A adrenergic receptor | ADRA1A |
| Alpha-1B adrenergic receptor | ADRA1B |
| Alpha-1D adrenergic receptor | ADRA1D |
| Alpha-2A adrenergic receptor | ADRA2A |
| Alpha-2B adrenergic receptor | ADRA2B |
| Alpha-2C adrenergic receptor | ADRA2C |
| Amine oxidase [flavin-containing] A | MAOA |
| Amine oxidase [flavin-containing] B | MAOB |
| Amyloid beta A4 protein | APP |
| Androgen receptor | AR |
| Antileukoproteinase | SLPI |
| Apoptosis regulator BAX | BAX |
| Apoptosis regulator Bcl-2 | BCL2 |
| Arachidonate 5-lipoxygenase | ALOX5 |
| Aryl hydrocarbon receptor | AHR |
| ATP synthase subunit beta, mitochondrial | ATP5F1B |
| ATP-binding cassette sub-family G member 2 | ABCA2 |
| Atrial natriuretic factor | ANF |
| Baculoviral IAP repeat-containing protein 4 | XIAP |
| Baculoviral IAP repeat-containing protein 5 | BIRC5 |
| Bcl-2-like protein 1 | BCL2L1 |
| Beta-1 adrenergic receptor | ADRB1 |
| Beta-2 adrenergic receptor | ADRB2 |
| Beta-lactamase | COA7 |
| Calcitonin receptor | CALCR |
| Calcium-activated potassium channel subunit alpha 1 | KCNMA1 |
| Calmodulin | CALM1 |
| cAMP-dependent protein kinase inhibitor alpha | PKIA |
| Carbonic anhydrase II | CA2 |
| Caspase-1 | CASP1 |
| Caspase-3 | CASP3 |
| Caspase-7 | CASP7 |
| Caspase-8 | CASP8 |
| Caspase-9 | CASP9 |
| Cathepsin D | CTSD |
| Caveolin-1 | CAV1 |
| C-C motif chemokine 2 | CCL2 |
| CD40 ligand | CD40LG |
| Cell division control protein 2 homolog | CDK1 |
| Cell division protein kinase 2 | CDK2 |
| Cell division protein kinase 4 | CDK4 |
| Cellular tumor antigen p53 | TP53 |
| CGMP-inhibited 3',5'-cyclic phosphodiesterase A | PDE3A |
| Chymotrypsinogen B | CTRB1 |
| Claudin-4 | CLDN4 |
| Coagulation factor VII | F7 |
| Coagulation factor Xa | F10 |
| Collagen alpha-1(I) chain | COL1A1 |
| Collagen alpha-1(III) chain | COL3A1 |
| C-reactive protein | CRP |
| C-X-C motif chemokine 10 | CXCL10 |
| C-X-C motif chemokine 11 | CXCL11 |
| C-X-C motif chemokine 2 | CXCL2 |
| Cyclin-A2 | CCNA2 |
| Cyclin-dependent kinase inhibitor 1 | CDKN1A |
| Cyclin-dependent kinase inhibitor 2A, isoforms 1/2/3 | CDKN2A |
| Cytochrome P450 1A1 | CYP1A1 |
| Cytochrome P450 1A2 | CYP1A2 |
| Cytochrome P450 1B1 | CYP1B1 |
| Cytochrome P450 3A4 | CYP3A4 |
| D(1B) dopamine receptor | DRD5 |
| D(2) dopamine receptor | DRD2 |
| DDB1- and CUL4-associated factor 5 | DCAF5 |
| Delta-type opioid receptor | OPRD1 |
| Dipeptidyl peptidase IV | DPP4 |
| DNA topoisomerase 1 | TOP1 |
| DNA topoisomerase 2-alpha | TOP2A |
| Dopamine D1 receptor | DRD1 |
| Dual oxidase 2 | DUOX2 |
| Dual specificity mitogen-activated protein kinase kinase 4 | MAP2K4 |
| E3 ubiquitin-protein ligase Mdm2 | MDM2 |
| Endothelin-1 | EDN1 |
| Endothelin-1 receptor | EDNRA |
| Endothelin-converting enzyme 1 | ECE1 |
| Epidermal growth factor receptor | EGFR |
| E-selectin | SELE |
| Estrogen receptor | ESR1 |
| Estrogen receptor beta | ESR2 |
| Estrogen sulfotransferase | SULT1E1 |
| ETS domain-containing protein Elk-1 | ELK1 |
| Eukaryotic translation initiation factor 6 | EIF6 |
| Fatty acid synthase | FASN |
| G1/S-specific cyclin-D1 | CCND1 |
| G2/mitotic-specific cyclin-B1 | CCNB1 |
| Gamma-aminobutyric acid receptor subunit alpha-1 | GABRA1 |
| Gamma-aminobutyric acid receptor subunit epsilon | GABRE |
| Gamma-aminobutyric acid receptor subunit gamma-3 | GABRG3 |
| Gamma-aminobutyric-acid receptor alpha-2 subunit | GABRA2 |
| Gamma-aminobutyric-acid receptor alpha-3 subunit | GABRA3 |
| Gamma-aminobutyric-acid receptor alpha-5 subunit | GABRA5 |
| Gamma-aminobutyric-acid receptor subunit alpha-6 | GABRA6 |
| Gap junction alpha-1 protein | GJA1 |
| Glucocorticoid receptor | NR3C1 |
| Glutamate receptor 2 | GRIA2 |
| Glutathione S-transferase Mu 1 | GSTM1 |
| Glutathione S-transferase Mu 2 | GSTM2 |
| Glutathione S-transferase P | GSTP1 |
| Glycogen phosphorylase, muscle form | PYGM |
| Glycogen synthase kinase-3 beta | GSK3B |
| Heat shock factor protein 1 | HSF1 |
| Heat shock protein beta-1 | HSPB1 |
| Heat shock protein HSP 90 | HSP90AA1 |
| Heme oxygenase 1 | HMOX1 |
| Hepatocyte growth factor receptor | MET |
| Hexokinase-2 | HK2 |
| Homeobox protein Nkx-3.1 | NKX3-1 |
| Hyaluronan synthase 2 | HAS2 |
| Hypoxia-inducible factor 1-alpha | HIF1A |
| Ig gamma-1 chain C region | IGHG1 |
| Induced myeloid leukemia cell differentiation protein Mcl-1 | MCL1 |
| Inhibitor of nuclear factor kappa-B kinase subunit alpha | CHUK |
| Inhibitor of nuclear factor kappa-B kinase subunit beta | IKBKB |
| Insulin receptor | INSR |
| Insulin-like growth factor II | IGF2 |
| Insulin-like growth factor-binding protein 3 | IGFBP3 |
| Integrin beta-3 | ITGB3 |
| Intercellular adhesion molecule 1 | ICAM1 |
| Interferon gamma | IFNG |
| Interferon regulatory factor 1 | IRF1 |
| Interleukin-1 alpha | IL1A |
| Interleukin-1 beta | IL1B |
| Interleukin-10 | IL10 |
| Interleukin-2 | IL2 |
| Interleukin-4 | IL4 |
| Interleukin-6 | IL6 |
| Interleukin-8 | IL8 |
| Interstitial collagenase | MMP1 |
| Kinetochore protein Nuf2 | NUF2 |
| Leukotriene A-4 hydrolase | LTA4H |
| Lysozyme | LYZ |
| Maltase-glucoamylase, intestinal | MGAM |
| Matrix metalloproteinase-9 | MMP9 |
| Metalloproteinase inhibitor 1 | TIMP1 |
| Microtubule-associated protein 2 | MAP2 |
| Mineralocorticoid receptor | NR3C2 |
| Mitogen-activated protein kinase 1 | MAPK1 |
| Mitogen-activated protein kinase 10 | MAPK10 |
| Mitogen-activated protein kinase 14 | MAPK14 |
| Mitogen-activated protein kinase 8 | MAPK8 |
| mRNA of PKA Catalytic Subunit C-alpha | PRKACA |
| mRNA of Protein-tyrosine phosphatase, non-receptor type 1 | PTPN1 |
| Muscarinic acetylcholine receptor M1 | CHRM1 |
| Muscarinic acetylcholine receptor M2 | CHRM2 |
| Muscarinic acetylcholine receptor M3 | CHRM3 |
| Muscarinic acetylcholine receptor M4 | CHRM4 |
| Muscarinic acetylcholine receptor M5 | CHRM5 |
| Mu-type opioid receptor | OPRM1 |
| Myc proto-oncogene protein | MYC |
| Myeloperoxidase | MPO |
| NAD(P)H dehydrogenase [quinone] 1 | NQO1 |
| NAD-dependent deacetylase sirtuin-1 | SIRT1 |
| NADH-ubiquinone oxidoreductase chain 6 | MT-ND6 |
| NADPH--cytochrome P450 reductase | POR |
| Neurofibromin | NF1 |
| Neuronal acetylcholine receptor protein, alpha-7 chain | CHRNA7 |
| Neuronal acetylcholine receptor subunit alpha-2 | CHRNA2 |
| Neutrophil cytosol factor 1 | NCF1 |
| NF-kappa-B inhibitor alpha | NFKBIA |
| Nitric oxide synthase, endothelial | NOS3 |
| Nitric oxide synthase, inducible | NOS2 |
| Nuclear factor erythroid 2-related factor 2 | NFE2L2 |
| Nuclear receptor coactivator 1 | NCOA1 |
| Nuclear receptor coactivator 2 | NCOA2 |
| Nuclear receptor subfamily 1 group I member 2 | NR1I2 |
| Nuclear receptor subfamily 1 group I member 3 | NR1I3 |
| Nucleophosmin | NPM1 |
| Ornithine decarboxylase | ODC1 |
| Osteopontin | SPP1 |
| Oxidized low-density lipoprotein receptor 1 | OLR1 |
| Peroxisome proliferator activated receptor delta | PPARD |
| Peroxisome proliferator activated receptor gamma | PPARG |
| Peroxisome proliferator-activated receptor alpha | PPARA |
| Phosphatidylinositol-3,4,5-trisphosphate 3-phosphatase and dual-specificity protein phosphatase PTEN | PTEN |
| Phosphatidylinositol-4,5-bisphosphate 3-kinase catalytic subunit, gamma isoform | PIK3CG |
| Pituitary adenylate cyclase-activating polypeptide | ADCYAP1 |
| Plasminogen activator inhibitor 1 | SERPINE1 |
| Poly [ADP-ribose] polymerase 1 | PARP1 |
| Poly [ADP-ribose] polymerase 4 | PARP4 |
| Potassium voltage-gated channel subfamily H member 2 | KCNH2 |
| Procollagen C-endopeptidase enhancer 1 | PCOLCE |
| Pro-epidermal growth factor | EGF |
| Progesterone receptor | PGR |
| Proliferating cell nuclear antigen | PCNA |
| Prostaglandin E synthase | PTGES |
| Prostaglandin E2 receptor EP3 subtype | PTGER3 |
| Prostaglandin G/H synthase 1 | PTGS1 |
| Prostaglandin G/H synthase 2 | PTGS2 |
| Prostatic acid phosphatase | ACPP |
| Proteasome assembly chaperone 1 | PSMG1 |
| Protein CBFA2T1 | RUNX1T1 |
| Protein kinase C alpha type | PRKCA |
| Protein kinase C beta type | PRKCB |
| Proto-oncogene c-Fos | FOS |
| Proto-oncogene serine/threonine-protein kinase Pim-1 | PIM1 |
| Puromycin-sensitive aminopeptidase | NPEPPS |
| RAC-alpha serine/threonine-protein kinase | AKT1 |
| RAF proto-oncogene serine/threonine-protein kinase | RAF1 |
| Ras association domain-containing protein 1 | RASSF1 |
| Ras GTPase-activating protein 1 | RASA1 |
| Receptor tyrosine-protein kinase erbB-2 | ERBB2 |
| Receptor tyrosine-protein kinase erbB-3 | ERBB3 |
| Retinoblastoma-associated protein | RB1 |
| Retinoic acid receptor RXR-alpha | RXRA |
| Retinoic acid receptor RXR-beta | RXRB |
| Runt-related transcription factor 2 | RUNX2 |
| Serine/threonine-protein kinase Chk1 | CHEK1 |
| Serine/threonine-protein kinase Chk2 | CHEK2 |
| Serine/threonine-protein phosphatase 2B catalytic subunit alpha isoform | PPP3CA |
| Serum paraoxonase/arylesterase 1 | PON1 |
| Signal transducer and activator of transcription 1-alpha/beta | STAT1 |
| Signal transducer and activator of transcription 3 | STAT3 |
| Sodium channel protein type 5 subunit alpha | SCN5A |
| Sodium-dependent dopamine transporter | SLC6A3 |
| Sodium-dependent noradrenaline transporter | SLC6A2 |
| Sodium-dependent serotonin transporter | SLC6A4 |
| Solute carrier family 2, facilitated glucose transporter member 4 | SLC2A4 |
| Stromelysin-1 | MMP3 |
| Superoxide dismutase [Cu-Zn] | SOD1 |
| Thrombin | F2R |
| Thrombomodulin | THBD |
| Tissue factor | F3 |
| Tissue-type plasminogen activator | PLAT |
| Transcription factor AP-1 | JUN |
| Transcription factor E2F1 | E2F1 |
| Transcription factor E2F2 | E2F2 |
| Transcription factor p65 | RELA |
| Transforming growth factor beta-1 | TGFB1 |
| Trypsin-1 | PRSS1 |
| Tumor necrosis factor | TNF |
| Type I iodothyronine deiodinase | DIO1 |
| Tyrosinase | TYR |
| Urokinase-type plasminogen activator | PLAU |
| Vascular cell adhesion protein 1 | VCAM1 |
| Vascular endothelial growth factor A | VEGFA |
| Vascular endothelial growth factor receptor 2 | KDR |
| Xanthine dehydrogenase/oxidase | XDH |

**References**

Liu, Y., Wang, Y., Ni, Y., Cheung, C.K.Y., Lam, K.S.L., Wang, Y., et al. (2020). Gut Microbiome Fermentation Determines the Efficacy of Exercise for Diabetes Prevention. *Cell Metab* 31(1)**,** 77-91 e75. doi: 10.1016/j.cmet.2019.11.001.
